# Complementary Superresolution Visualization of Composite Plant Microtubule Organization and Dynamics

**DOI:** 10.3389/fpls.2020.00693

**Published:** 2020-06-05

**Authors:** Tereza Vavrdová, Pavel Křenek, Miroslav Ovečka, Olga Šamajová, Pavlína Floková, Petra Illešová, Renáta Šnaurová, Jozef Šamaj, George Komis

**Affiliations:** Department of Cell Biology, Centre of the Region Haná for Biotechnological and Agricultural Research, Faculty of Science, Palacký University Olomouc, Olomouc, Czechia

**Keywords:** Airyscan confocal laser scanning microscopy, microtubule associated proteins, microtubules, photoactivation localization microscopy, photoconvertible protein, single molecule localization microscopy, structured illumination microscopy

## Abstract

Microtubule bundling is an essential mechanism underlying the biased organization of interphase and mitotic microtubular systems of eukaryotes in ordered arrays. Microtubule bundle formation can be exemplified in plants, where the formation of parallel microtubule systems in the cell cortex or the spindle midzone is largely owing to the microtubule crosslinking activity of a family of microtubule associated proteins, designated as MAP65s. Among the nine members of this family in *Arabidopsis thaliana*, MAP65-1 and MAP65-2 are ubiquitous and functionally redundant. Crosslinked microtubules can form high-order arrays, which are difficult to track using widefield or confocal laser scanning microscopy approaches. Here, we followed spatiotemporal patterns of MAP65-2 localization in hypocotyl cells of Arabidopsis stably expressing fluorescent protein fusions of MAP65-2 and tubulin. To circumvent imaging difficulties arising from the density of cortical microtubule bundles, we use different superresolution approaches including Airyscan confocal laser scanning microscopy (ACLSM), structured illumination microscopy (SIM), total internal reflection SIM (TIRF-SIM), and photoactivation localization microscopy (PALM). We provide insights into spatiotemporal relations between microtubules and MAP65-2 crossbridges by combining SIM and ACLSM. We obtain further details on MAP65-2 distribution by single molecule localization microscopy (SMLM) imaging of either mEos3.2-MAP65-2 stochastic photoconversion, or eGFP-MAP65-2 stochastic emission fluctuations under specific illumination conditions. Time-dependent dynamics of MAP65-2 were tracked at variable time resolution using SIM, TIRF-SIM, and ACLSM and post-acquisition kymograph analysis. ACLSM imaging further allowed to track end-wise dynamics of microtubules labeled with TUA6-GFP and to correlate them with concomitant fluctuations of MAP65-2 tagged with tagRFP. All different microscopy modules examined herein are accompanied by restrictions in either the spatial resolution achieved, or in the frame rates of image acquisition. PALM imaging is compromised by speed of acquisition. This limitation was partially compensated by exploiting emission fluctuations of eGFP which allowed much higher photon counts at substantially smaller time series compared to mEos3.2. SIM, TIRF-SIM, and ACLSM were the methods of choice to follow the dynamics of MAP65-2 in bundles of different complexity. Conclusively, the combination of different superresolution methods allowed for inferences on the distribution and dynamics of MAP65-2 within microtubule bundles of living *A. thaliana* cells.

## Introduction

Microtubules are essential components of the plant cytoskeleton and are crucial for fundamental cellular functions, including cell division, growth and morphogenesis (reviewed in Panteris and Galatis, 2005; Yamada and Goshima, 2017; Eng and Sampathkumar, 2018; Lazzaro et al., 2018; Sapala et al., 2018). Higher plants are devoid of a structurally discernible microtubule organizing center (MTOC), therefore formation and organization of plant-unique microtubule arrays, such as the interphase cortical system, the premitotic preprophase microtubule band, the acentrosomal mitotic spindle and the cytokinetic phragmoplast, rely on interactions between microtubules and several microtubule associated proteins (MAPs) with diverse functions (Bannigan et al., 2008; Müller et al., 2009; Lee and Liu, 2013; Buschmann and Zachgo, 2016; Smertenko, 2018).

Such proteins are involved in the spatiotemporal control of microtubule nucleation (e.g., Walia et al., 2014; Nakamura, 2015; Tian and Kong, 2019), the regulation of end-wise microtubule dynamics (e.g., Nakamura et al., 2018; Lindeboom et al., 2019), microtubule clearance via severing (e.g., Nakamura et al., 2010; Tulin et al., 2012; Deinum et al., 2017; Wang et al., 2018), the formation of higher order microtubule assemblies via physical microtubule crosslinking (e.g., Ma et al., 2016; Molines et al., 2018; Burkart and Dixit, 2019), or the adjustment of microtubule positioning by different microtubule-dependent motor activities (e.g., Zhu et al., 2015; Oda, 2018). A particular group of proteins associated with microtubules, are those with dual affinity for both the microtubule and the actin filament surface (reviewed in Schneider and Persson, 2015; Krtková et al., 2016). Notable examples include members of the plant FORMIN family (e.g., Sun et al., 2017; Wu and Bezanilla, 2018; Kollárová et al., 2020), members of the ARP2/3 actin nucleation complex (Havelková et al., 2015) and motor proteins of either the kinesin or the myosin superfamilies (e.g., Schneider and Persson, 2015). In this respect kinesins with calponin homology domains such as tobacco KCH1 or cotton KCH2 (e.g., Xu et al., 2009; Buschmann et al., 2010), were found to bind to both cytoskeletal filaments and especially in the case of KCH2, to crosslink actin and microtubules (Xu et al., 2009). Likewise, some plant myosins have been found to colocalize with microtubular structures, such as the mitotic spindle (e.g., Sun et al., 2018 for MYOSIN XI) or to directly interact with microtubules (Wu and Bezanilla, 2014 for MYOSIN VIII).

The plant interphase cortical array is a widespread microtubule system lying at the close vicinity of the plasma membrane, and it is intimately associated with cell growth and differentiation (Elliott and Shaw, 2018b). It can promptly reorganize in response to physical (reviewed in Lindeboom et al., 2013; Nakamura, 2015; Hamant et al., 2019), or hormonal (Vineyard et al., 2013; Elliott and Shaw, 2018a; Adamowski et al., 2019; True and Shaw, 2020) signals, in order to redefine cell growth directionality by blueprinting the orientation of cellulose deposition in the overlying cell wall (Chen et al., 2016).

The directional growth of plant cells requires cortical microtubules of uniform orientation. At large, this is achieved through microtubule interactions mediated by MAPs. Symmetry breaking in the cortical array arises from several different mechanisms, which include the spatial control of microtubule nucleation (Lindeboom et al., 2013), or microtubule severing (reviewed in Luptovčiak et al., 2017), the tight regulation of plus-end (Galva et al., 2014; Lindeboom et al., 2019), or minus-end (Nakamura et al., 2018) stability and dynamics, and the bundling or elimination of microtubules that encounter each other during their end-wise dynamic length fluctuations (Dixit and Cyr, 2004; Wightman and Turner, 2007; Zhang et al., 2013).

Based on the angle of contact, microtubule encounters may have either a constructive, or destructive outcome, leading to sustained microtubule growth at the preferred orientation, or initiating a catastrophe event eliminating microtubules of the unfavorable orientation (Wightman and Turner, 2007; Zhang et al., 2013). The outcome of microtubule convergence depends on the angle of encounter (Chi and Ambrose, 2016). If the angle is greater than 40°, the encounter results in either a catastrophe (a rapid shrinkage initiated at the tip of the microtubule that touches the lattice of another), or a crossover, where katanin-mediated severing may selectively occur and cleave one of the two microtubules (Wightman and Turner, 2007; Zhang et al., 2013). When the angle of encounter is less than 40°, the microtubules tend to co-align and bundle (Dixit and Cyr, 2004) by means of physical crosslinking via MAPs, leading eventually to the formation of a biased array with predominant orientation and parallel microtubule arrangement (van Damme et al., 2004).

From *in vitro* studies based on MAP65-1, it was shown that it can exist in a monomeric state capable of coating individual microtubules, being able to dimerize in a “zippering” process and construct 25 nm cross bridges when two antiparallel microtubules come in close contact (Gaillard et al., 2008; Tulin et al., 2012). In sharp contrast, the human MAP65 homolog PRC1 (Protein Regulator of Cytokinesis 1) and likewise the fission yeast homolog Ase1p (Anaphase spindle elongation 1), form obligate dimers or homotetramers. In this case, formation of cross bridge depends on the flexibility of the oligomeric molecule, which assumes a rigid structure when confined in the overlap of two antiparallel microtubules (Subramanian et al., 2010).

Regardless of the mechanisms leading to their crosslinking, microtubule bundling is essential for the building of universal microtubule systems, such as the premitotic preprophase microtubule band and the interzonal telophase system. Moreover, the coalescence of adjacent microtubules to tight bundles was shown to be related to the morphogenesis of particular cell types with unique cell wall patterning such as differentiating tracheary (Mao, 2006; Pesquet et al., 2010; Derbyshire et al., 2015) and protoxylem elements (Schneider et al., 2020).

Apart from such developmental processes, environmental factors and hormones also induce symmetry breaking in the cortical array (e.g., Elliott and Shaw, 2018b; True and Shaw, 2020). Although katanin-mediated severing was already shown to have a major contribution in the induction of uniform microtubule orientation (e.g., Chen et al., 2014; Sassi et al., 2014; reviewed in Luptovčiak et al., 2017), the role of bundling by means of physical crosslinking of adjacent microtubules has not been addressed extensively.

From the MAPs related to the formation of microtubule bundles, the MAP65 family is the best characterized. Based on transmission electron micrographs, MAP65 proteins exemplified by MAP65-1 form 25 nm crossbridges between adjacent antiparallel microtubules (Chan et al., 1999; Tulin et al., 2012). Arabidopsis MAP65s bind to microtubules via C-terminal domain-located binding sites, and their function depends on the formation of homodimers via their N-terminal domain (Smertenko et al., 2004). For MAP65-1, MAP65-2, and MAP65-5, it has been shown that they do not promote microtubule polymerization, yet they slow down depolymerization rates (van Damme et al., 2004; Lucas et al., 2011). Moreover, MAP65-1 was recently shown to prohibit katanin from binding to microtubule bundles, thus protecting them from severing (Burkart and Dixit, 2019).

In Arabidopsis, nine genes belonging to this family were identified (Hussey et al., 2002), with different subcellular localizations, expression patterns throughout cell cycle (van Damme et al., 2004) and responses to in/activation during cell cycle (Smertenko et al., 2006; Boruc et al., 2017). Several MAP65s were observed to colocalize with mitotic microtubule arrays, specifically MAP65-1 (Smertenko et al., 2004), MAP65-2 (Lucas and Shaw, 2012), MAP65-3 (Caillaud et al., 2008; Ho et al., 2012; Vavrdová et al., 2019b), MAP65-4 (van Damme et al., 2004), MAP65-5 and MAP65-6 (Smertenko et al., 2008), with some of them being involved in the progression of mitosis (Beck et al., 2010, 2011; Sasabe et al., 2011; Li et al., 2017; Vavrdová et al., 2019a). Particularly in this case, the phosphorylation of MAP65s at their C-terminal domain via MITOGEN ACTIVATED PROTEIN KINASE 4 (MPK4) and MPK6 (Beck et al., 2010; Kosetsu et al., 2010; Sasabe et al., 2011), cyclin dependent kinases (Smertenko et al., 2006) and Aurora kinases, is a universal negative regulation of their affinity for the microtubule surface (reviewed in Komis et al., 2011; Vavrdová et al., 2019a). More importantly, MAP65-1, MAP65-2 and MAP65-5 colocalize with cortical microtubules (van Damme et al., 2004; Lucas et al., 2011). Owing to their functional redundancy, co-expression and spatiotemporal colocalization (Lucas and Shaw, 2012), single mutants of either *MAP65-1* or *MAP65-2* do not show a discernible phenotype. However, in double *map65-1 map65-2* mutants an overall growth retardation was observed (Lucas et al., 2011).

Microtubule bundles represent a crowded environment hindering the possibilities to track the dynamic behavior of individual components with diffraction limited microscopy approaches. In a previous study, we demonstrated the capacity of structured illumination microscopy (SIM) to delineate the microtubule content of complex bundles in the cell cortex, and within the limitations of the method, to record dynamics of individual microtubules at accepted frame rates (Komis et al., 2014). In the present study, we extend this paradigm to follow the distribution and the dynamics of a universal microtubule crosslinking MAP, either alone or in parallel, to appropriately labeled microtubules.

For this reason, we employ different superresolution microscopy methods to extrapolate information on the organization and the dynamics of MAP65-2 in living *A. thaliana* hypocotyl epidermal cells, expressing appropriate fluorescent protein markers. We use Airyscan confocal laser scanning microscopy (ACLSM) and 2D SIM to obtain high resolution images of fluorescently labeled MAP65-2 and its association with cortical microtubule bundles. Dynamic properties of MAP65-2 are deciphered at different time scales using ACLSM, 2D SIM and total internal reflection (TIRF) SIM. Finally, the specific arrangement of MAP65-2 molecules is approached by single molecule photoactivation localization microscopy (PALM) from either the stochastic photoconversion of a mEos3.2-MAP65-2 molecular marker, or by calculating stochastic optical fluctuations of an eGFP-MAP65-2 fusion protein under specific illumination conditions.

## Materials and Methods

### Plant Material

*Arabidopsis thaliana* (L.) was used for all experiments presented herein. Stably transformed Arabidopsis lines carrying *proMAP65-2:eGFP:MAP65-2*, *proMAP65-2:tagRFP:MAP65-2*, and *proMAP65-2:mEos3.2:MAP65-2* constructs, were prepared in a wild type ecotype Columbia (Col-0) background. Other Arabidopsis lines were stably transformed with *proCaMV35S:TUA6:GFP* or *proUBQ1:mRFP:TUB6* constructs and for colocalization purposes such lines were crossed with plants expressing appropriate MAP65-2 markers. Plants were grown on Phytagel (Sigma, Czechia) solidified half-strength Murashige–Skoog (1/2 MS) medium supplemented with 1% (w/v) sucrose and under controlled environmental conditions (Beck et al., 2010).

### Transgenic Plant Construction

Constructs for N-terminal fluorescent protein fusions of MAP65-2 were prepared using binary vector pGWB502link (Vavrdová et al., 2019b), a modified version of original destination vector pGWB502 (Nakagawa et al., 2007). All primers used for cloning are listed in Supplementary Table S1. PCR product (2616 bp) corresponding to the native promoter region of *MAP65-2* gene was obtained using PCR with primers pMAP65-2-F and pMAP65-2-R, which contain *Pac*I and *Acc*65I restriction site, respectively, and template genomic DNA isolated from *A. thaliana* Col-0. This PCR product was double-digested with *Pac*I and *Acc*65I and ligated into vector pGWB502link, also double-digested with *Pac*I and *Acc*65I, to generate construct pGWB502link-*proMAP65-2*. An open reading frame (ORF) of *MAP65-2* including stop codon was amplified from cDNA (isolated from *A. thaliana* Col-0) using primers MAP65-2cDNA-F and MAP65-2cDNA-R, which contain *Acc*65I and *Bsi*WI restriction site, respectively. PCR product encompassing ORF of *MAP65-2* was double-digested with *Acc*65I and *Bsi*WI and ligated into *Acc*65I digested pGWB502link-*proMAP65-2* to generate construct proGWB502link-*proMAP65-2:MAP65-2*. *Acc*65I and *Bsi*WI are isocaudomers producing the same sticky ends. Therefore, pGWB502link-*pMAP65-2:MAP65-2* contains only a single *Acc*65I restriction site in between *pMAP65-2* region and start codon of *MAP65-2* ORF. Coding region of *EGFP* was amplified with primers eGFP-F and eGFP-R, whereas coding region of *tagRFP* was amplified with primers tagRFP-F and tagRFP-R. All four primers contain a single *Acc*65I restriction site near their 5′-end. In addition, stop codon in the sequence of reverse primers is replaced with in frame stretch of nucleotides coding for a linker (EAAAK)_3_ (Werner et al., 2006), which interconnects fluorescent protein tags with MAP65-2. PCR products containing coding regions of *EGFP* and *tagRFP* were digested with *Acc*65I and ligated into *Acc*65I digested pGWB502link-*proMAP65-2:MAP65-2* to generate constructs *proMAP65-2:EGFP:MAP65-2* and *proMAP65-2:tagRFP:MAP65-2*, respectively. To prepare the construct *proMAP65-2:mEos3.2:MAP65-2*, coding sequence of *mEos3.2* (Zhang et al., 2012) was optimized for expression in *A. thaliana* by GeneOptimizer^TM^ software (Thermo Fisher Scientific, United States) and synthetized by GeneArt^TM^ (Thermo Fisher Scientific, United States). In the 5′ to 3′ direction synthetic *mEos3.2* DNA fragment contains *Acc*65I restriction site upstream of the start codon, codon optimized ORF of *mEos3.2*, DNA sequence for (EAAAK)_3_ linker replacing stop codon and *Acc*65I restriction site immediately following linker encoding sequence. Synthetic *mEos3.2* fragment was digested with *Acc*65I and ligated into *Acc*65I digested construct pGWB502link-*proMAP65-2:MAP65-2*. All prepared constructs were verified by Sanger sequencing and used for the preparation of stably transformed *A. thaliana* Col-0 ecotype transgenic plants as described before (Vavrdová et al., 2019b).

Positive T1 seedlings (i.e., the first generation following transformation) were selected either on the basis of antibiotic resistance, or upon detection of fluorescence. Few positive lines, showing similar fluorescence intensity, were chosen for subsequent propagation for each construct. In the T3 generation of selection, at least one homozygous line showing uniform fluorescence intensity was obtained for each construct. Fluorescence intensity was inspected at the root apex at the seedling stage, because the root apex is devoid of autofluorescence in both channels used for the selection (green channel for eGFP-MAP65-2, TUA6-GFP and unconverted mEos3.2-MAP65-2 and red channel for mRFP-TUB6, tagRFP-MAP65-2 and converted mEos3.2-MAP65-2). All the experiments were performed on T3 generation seedlings of one selected homozygous line for each construct. Functionality of all constructs was deduced by the fact that all transgenic seedlings, had no discernible phenotype and during microscopy exhibited the expected localization patterns of MAP65-2, either when visualized alone, or together with appropriately labeled microtubules.

### Sample Preparation

Seedlings grown for 3–4 days after germination were selected according to the expression of constructs under an epifluorescence microscope. Selected seedlings were transferred on a microscopic slide (containing a spacer from double-sided sticky tape) into liquid 1/2 MS medium, and after applying a coverslip, parafilm was used to gently seal the sample at the margins of the coverslip, in order to prevent evaporation of medium and to stabilize samples for microscopic observation. When the 100 × /1.57 NA oil-immersion objective was used, samples were prepared within Attofluor cell chambers (Invitrogen, United States) and sandwiched between a high-precision and low-thickness-tolerance Nexterion round coverslip (facing the objective; coverslip thickness (D) = 0.17 ± 0.003 mm, diameter = 25 mm; Schott, Czech Republic; Komis et al., 2014, 2015), and a common 18 mm OD round coverslip of the same thickness. For TIRF-SIM imaging, 3–4 days old seedlings were secured in Attofluor cell chambers, embedded in 1% (w/v) low gelling temperature agarose dissolved in liquid 1/2 MS medium.

### Microscopy Setup and Acquisition

For this study, following microscopes were used: AxioObserver LSM 880 with Airyscan (ACLSM; Carl Zeiss, Germany), AxioImager Z.1 equipped with the Elyra PS.1 superresolution system supporting the SIM and PALM/STORM module (Carl Zeiss, Germany) and a custom built TIRF-SIM microscope maintained in the Advanced Imaging Center of Janelia Research Campus (Kner et al., 2009; Ashburn, United States). With the ACLSM, either 40×/1.40 NA, or 63×/1.40 NA, oil-immersion, Plan Apochromat objectives were used with appropriate oil (Immersol 518F with refractive index of 1.518). Single photon excitation laser lines were used throughout with the 488 nm line for GFP excitation and 561 nm for mRFP and tagRFP excitation. Appropriate beam splitters and emission fluorescence filter blocks (BP420-480+BP495-550 for GFP detection and BP495-550 + LP570 for mRFP and tagRFP detection) were used in ACLSM and signal was detected by a 32 GaAsP detector with fully opened pinhole. Samples were scanned with the super-resolution mode of the ACLSM allowing optimum resolution for acquired single Z-stacks or time-lapsed 2D acquisitions. Owing to the effective light-collecting capacity of the ACLSM and the sensitivity of the GaAsP detector the laser power at the excitation was set to a level not exceeding 2% of the range available. Acquired data were analyzed with Zen 2014 software (Blue Version; Carl Zeiss, Germany).

For SIM acquisitions with the Zeiss Elyra PS.1 platform, either 63×/1.40 NA or 100×/1.57 NA, oil-immersion, Alpha Plan Apochromat objectives were used with Immersol 518F and Immersol HI (with refractive index of 1.66), respectively. Samples were illuminated with a 488 nm laser line for eGFP excitation and a 561 nm laser line for mRFP or tagRFP excitation. For eGFP a BP495-575/LP750 filter was used while for mRFP or tagRFP a BP570-620/LP750 was used. Recordings were done using a PCO.Edge 5.5 sCMOS camera. For highest resolution possible samples were illuminated with five rotations and five phase steps, while for time lapsed imaging rotations of the patterned light were restricted to three (Komis et al., 2014, 2015). Reconstruction of SIM images and generation of kymographs were done using Zen software with appropriate licenses. The TIRF-SIM recordings were done with a custom built system and house written software as previously published (Kner et al., 2009). Owing to the setup of sample preparation we obtained very stable recordings over periods between 10–30 min, without loss of focus.

PALM localizations were performed on the dedicated Elyra PS.1 microscopy platform. Photoconversion of the green emitter form of mEos3.2 (excited with a 488 nm laser line and visualized through a BP420-480/BP495-560/LP650 dual bandpass/longpass filter) to the red emitter form, was done using a 405 nm laser line and a 561 nm excitation line. For simultaneous activation and excitation of the photoconverting form of mEos3.2 (i.e., molecules converting to the red emitter form), a BP420-480/BP570-640/LP740 dual bandpass/longpass filter was used. Photons were collected by the EM CCD sensor of an Andor iXon 897 Ultra camera without EM gain. Illumination of the sample was done using either the Highly Inclined and Laminated Optical Sheet regime (HILO; Tokunaga et al., 2008), or the ultra-high power TIRF mode of the system. For improving precision of localization, photons of stochastically photoconverting mEos3.2 molecules, were captured in time series experiments of 5,000 – 10,000 time points with exposure times ranging between 40 ms and 80 ms. The readout was processed using the PALM module of Zen software and since localization was done in 2D, overlapping localizations were discarded. In the example given in the appropriate section, results are presented both collectively and for representative examples of individual localizations.

Occasionally, more conventional fluorophores such as GFP and its variants, eGFP and YFP, may exhibit fluorescence intensity fluctuations under special conditions of excitation at or out of their nominal excitation wavelength (reviewed in Bagshaw and Cherny, 2006). For example, eGFP was shown to blink at acidic pH values when illuminated with a 405 nm laser (Haupts et al., 1998) or it may exhibit oxidative photoconversion to a red emitter following excitation with a 532 nm laser line (Sen et al., 2019). The versatility of more conventional fluorophores in SMLM has led to the development of unique methods including Stochastic Optical Fluctuations Imaging (SOFI; Dertinger et al., 2009) or Bayesian analysis of blinking and bleaching (3B; Cox et al., 2012) with a very big potential to broaden SMLM applications in plants.

Optical fluctuations of eGFP, were recorded in a similar way as described for mEos3.2, by illuminating the sample simultaneously with the 405 nm and the 488 nm laser lines of Elyra PS.1, using the BP420-480/BP495-560/LP650 dual bandpass/longpass filter. The use of 405 nm illumination was previously shown to promote emission fluctuations of eGFP and was used accordingly (Marcus and Raulet, 2013). Better photon counts were achieved under the ultra high power TIRF mode compared to HILO illumination.

### Image Processing and Quantitative Analysis

Raw ACLSM and SIM images were acquired with Zen software. Fluorescence intensity profiling was performed as described previously (Komis et al., 2014). Briefly, intensity profiles were measured directly in Zen software. Raw values were exported to Microsoft Excel (Microsoft, United States), normalized to a range between 0 and 1 and plotted against distance. These scatterplots were used to measure full-width at half maximum (FWHM) with Image J (Schneider et al., 2012). TIRF-SIM images were obtained and processed with custom-written software (Kner et al., 2009). In this case, fluorescence intensity profiles were measured in Image J, then previously described workflow was followed (Komis et al., 2014).

Similarly, for analysis of microtubule bundles and differential distribution of MAP65-2 along microtubules, both perpendicular and longitudinal fluorescence intensity profiles were done in Zen software. Due to the fact that MAP65-2 decorates microtubules in a discontinuous manner, for each perpendicular fluorescence intensity measurement five profiles were drawn and their values were averaged. Altogether, eight sets (consisting of measurements of a bundle and two branches stemming from it) of perpendicular profiles were made. After exporting raw values to Microsoft Excel, data were normalized and plotted against distance.

Pearson’s (Dunn et al., 2011) and Manders’ (Manders et al., 1992) correlation coefficients denoting the extend of colocalization between tagged microtubules and MAP65-2 protein fusions, were automatically extrapolated by means of the colocalization tool of the licensed version of Zen software. Colocalizations were automatically thresholded according to Costes (Costes et al., 2004).

From time-lapsed images taken by ACLSM and SIM, kymographs were generated with the appropriate plugin of Zen software (Blue version). For generating kymographs of TIRF-SIM acquisitions, we instead used the Multi Kymograph plugin of Image J^1^. Angles and distances needed for calculations were measured in ImageJ. Parameters describing MAP65-2 dynamics were calculated as described previously (Smal et al., 2010). Briefly, growth rates were calculated by correcting the tangent values of slopes corresponding to growth or shrinkage phases, with the pixel size and the frame rate of each respective acquisition. Measures deduced from kymographs included plus-end growth and shrinkage rates and catastrophe/rescue frequencies and time spent in growth and shrinkage. Catastrophe rates were deduced by dividing the sum of shrinkage onset events observed by the total amount of time spent in growth (extension) phases, while rescue frequencies were deduced by dividing the sum of growth onset events observed by the total amount of time spent in shrinkage (retraction) phases (Gardner et al., 2013; Kapoor et al., 2019). Briefly the equations used in this case can be formulated as:

$$f_{c\,a\,t}=\frac{N_{c\,a\,t}}{\Sigma \,t_{g\,r\,o\,w\,t\,h}}$$

for the calculation of catastrophe frequencies, where *f*_*cat*_ is the catastrophe frequency, *N*_*cat*_ is the total number of catastrophes measured and *Σt_*growth*_* is the total time spent in growth, while similarly the calculation of rescue frequency is based on the formula:

$$f_{r\,e\,s}=\frac{N_{r\,e\,s}}{\Sigma \,t_{s\,h\,r\,i\,n\,k\,a\,g\,e}}$$

where *f*_*res*_ is the rescue frequency, *N*_*res*_ is the total number of rescues measured and *Σt_*shrinkage*_* is the total time spent in shrinkage.

### Statistical Analysis

For statistical analysis, all datasets were first tested for normality of data distribution by means of Shapiro–Wilk test. Based on the results of Shapiro–Wilk test, either unpaired two-sample *t*-tests or Mann–Whitney *U* tests were performed. All tests were calculated in STATISTICA (version 13.4.0.14; Statsoft, United States). Statistical significance was inferred according to the calculated *p*-values.

## Results

### Superresolution of MAP65-2 Decorating Microtubules

The purpose of our study, was to report the fine structure of microtubule bundles and track the organization and dynamics of fluorescently tagged MAP65-2 within such bundles. We chose for this purpose three methods, namely, ACLSM, widefield 2D SIM and TIRF-SIM, which were initially characterized in terms of spatial resolution at the settings used for time-lapsed imaging. As previously mentioned, the quantitative measure in this case was the FWHM of normalized fluorescence intensity linear profiles encompassing the entire microtubule or MAP65-2 signal width.

**FIGURE 1:**
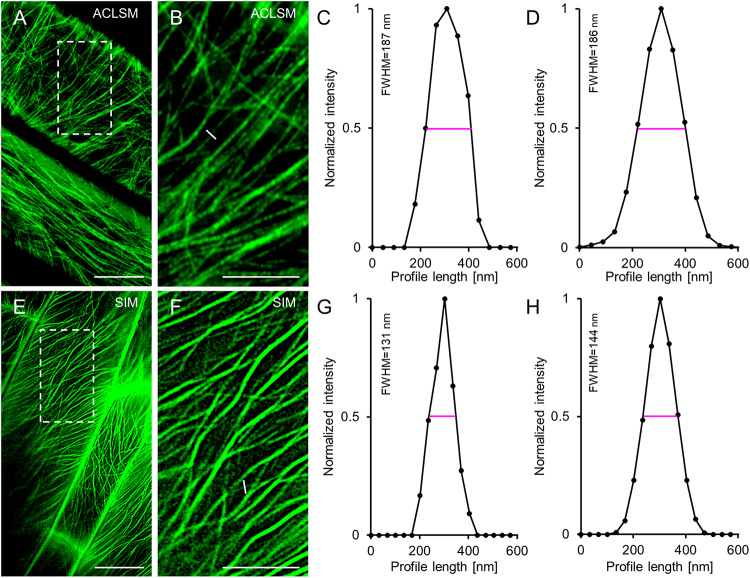
Comparison of ACLSM and SIM in resolving details of TUA6-GFP labeled cortical microtubules in hypocotyl epidermal cells of Arabidopsis labeled. **(A)** Overview of image from ACLSM (objective 63×/1.40 NA). **(B)** Magnified view of the boxed area in **(A)**. The white line corresponds to a perpendicular profile, for normalized intensity measurement. **(C)** Graph depicting normalized fluorescence intensity corresponding to the profile shown in **(B)**. **(D)** Averaged, coaligned, and normalized intensity profiles of individual TUA6-GFP-labeled microtubules visualized by ACLSM (*N* = 43; FWHM – full-width at half maximum). **(E)** Overview of image from 2D-SIM (objective 63×/1.40 NA). **(F)** Magnified view of the boxed area in **(E)**. The white line in **(F)** shows a profile, used for normalized intensity measurement. **(G)** Representative plot of a normalized fluorescence intensity corresponding to the profile drawn in **(F)**. **(H)** Averaged, coaligned, and normalized intensity profiles of individual TUA6-GFP-labeled microtubules visualized with 2D-SIM (*N* = 44). Scale bars = 10 μm **(A,E)**, or 5 μm **(B,F)**, respectively.

**FIGURE 2:**
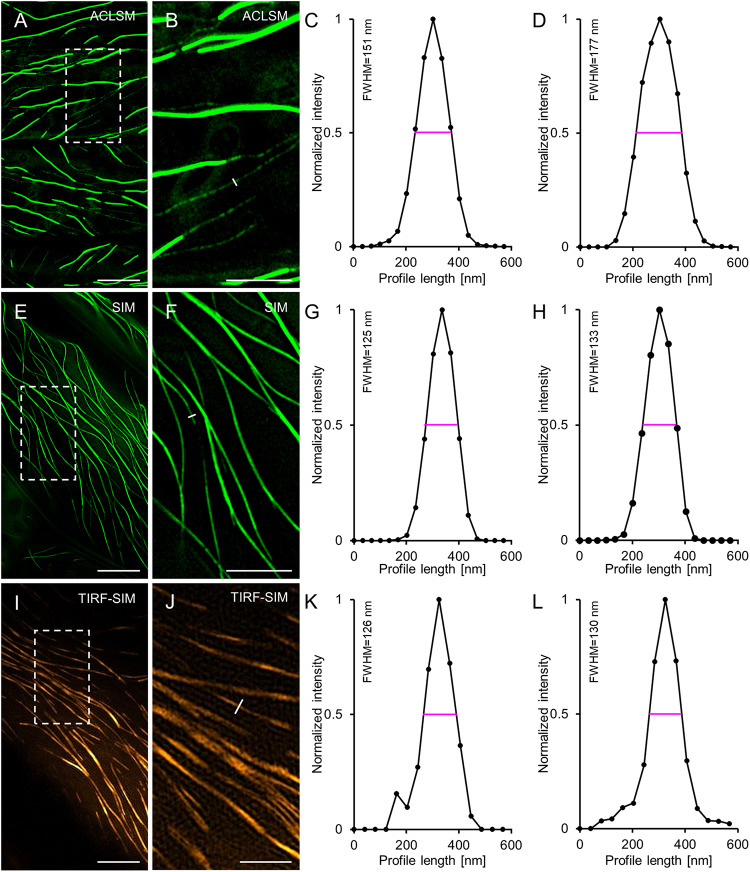
Comparison of ACLSM, SIM and TIRF-SIM in resolving MAP65-2 decoration of cortical microtubules in hypocotyl epidermal cells of Arabidopsis expressing eGFP-MAP65-2. **(A)** ACLSM overview. **(B)** Close-up image of the boxed area of **(A)** with profile used for normalized intensity measurement (white line). **(C)** Representative quantitative depiction of normalized fluorescence intensity of the individual profile drawn in **(B)**. **(D)** Averaged, coaligned, and normalized intensity profiles of basal MAP65-2 decoration of microtubules (*N* = 36; FWHM, full-width at half maximum). **(E)** 2D-SIM overview (objective 63×/1.40 NA). **(F)** Magnified view of boxed area in **(E)** with the perpendicular profile, for normalized intensity measurement (white line). **(G)** Graph depiction of the normalized intensity corresponding to the profile drawn in **(F)**. **(H)** Averaged, coaligned, and normalized intensity profiles of basal MAP65-2 decoration of microtubules (*N* = 39). **(I)** Overview image from TIRF-SIM (objective 100×/1.49 NA). **(J)** Magnification of the boxed area of **(I)**. **(K)** Normalized intensity of the perpendicular profile drawn in **(J)**. **(L)** Averaged, coaligned, and normalized intensity profiles of basal MAP65-2 decoration of microtubules visualized with TIRF-SIM (*N* = 55). Scale bars = 10 μm **(A,E)**, 5 μm **(B,F,I)**, and 2 μm **(J)** respectively.

When comparing the resolution of TUA6-GFP-labeled individual microtubules visualized by means of ACLSM (Figures 1A,B) or 2D SIM (Figures 1E,F), we found significant differences especially after defining the FWHM of normalized intensity profiles (Figures 1C,D cf. Figures 1G,H) using in both cases a 63×/1.40 NA oil immersion objective. Quantitatively the resolution of individual microtubules averaged at 186 ± 27 nm (mean ± SD; *N* = 43, Figure 1D) with ACLSM and at 144 ± 25 nm (mean ± SD; *N* = 44, Figure 1H) with 2D SIM. This difference is statistically significant (*t*-value = −7.1839; *p* < 0.001). However the FWHM value reported for 2D SIM was higher for the 63 × /1.40 NA objective than that reported previously (Komis et al., 2014) owing to the use of lower Wiener filter during image reconstruction to compensate for the lower signal to noise ratio of the hypocotyl cells used herein.

**FIGURE 3:**
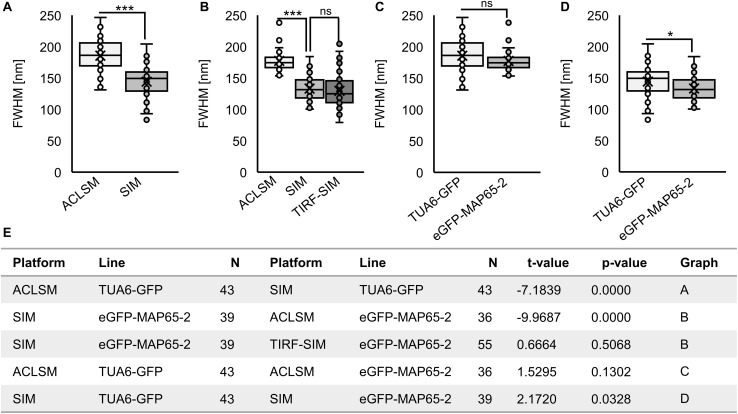
Quantitative analysis of resolution achieved by using ACLSM, SIM, and TIRF-SIM in resolving details of cortical microtubules (MTs) with TUA6-GFP marker or eGFP-MAP65-2 decoration of cortical MTs. Boxplots present the full-width at half maximum values (FWHM). **(A)** Comparison of ACLSM and SIM in resolving MTs with TUA6-GFP marker. **(B)** Comparison of ACLSM, SIM, and TIRF-SIM in resolving eGFP-MAP65-2 decoration of MTs. **(C)** Resolution reached by ACLSM for either TUA6-GFP or eGFP-MAP65-2. **(D)** Resolution reached by SIM for either TUA6-GFP or eGFP-MAP65-2. **(A–E)** Unpaired two-sample *t*-tests were used for statistical analysis (*** significant at *p* 0.001, * significant at *p* 0.05, ns, not statistically significant) and the results are in **(E)**. Description of box plot: average is presented by ×, median by the middle line, 1st quartile by the bottom line, 3rd quartile by the top line; the whiskers lie within the 1.5 × interquartile range (defined from the 1st to the 3rd quartile), outliers are marked by points.

As 2D SIM and TIRF-SIM were used for time lapsed recordings of eGFP-MAP65-2, we compared their resolution potential in cells expressing eGFP-MAP65-2 and referenced them against ACLSM. In this case, eGFP-MAP65-2 decorated microtubules were resolved by ACLSM (Figures 2A–C) at 177 ± 19 nm (FWHM of normalized intensity profiles; mean ± SD; *N* = 36, Figure 2D). For 2D SIM (Figures 2E–G) and TIRF-SIM (Figures 2I–K) the resolution of eGFP-MAP65-2 decorated microtubules was considerably improved (133 ± 20 nm; mean ± SD; *N* = 39, Figure 2H for SIM; 130 ± 28 nm; mean ± SD; *N* = 55, Figure 2L for TIRF-SIM). As denoted, 2D SIM and TIRF-SIM showed significantly better resolution compared to ACLSM with either TUA6-GFP (Figures 3A,E) or eGFP-MAP65-2 (Figures 3B,E), without showing differences when compared to each other (Figures 3B,E). Similarly, when comparing the resolution of the two labeled structures, we did not find considerable difference between them neither by ACLSM (Figures 3C,E) or by 2D SIM (Figures 3D,E).

For dual channel visualization of both microtubules and MAP65-2, it was necessary to analyze the resolution of red tags for both microtubules (mRFP-TUB6; Supplementary Figures S1A–C) and MAP65-2 (tagRFP-MAP65-2) when visualized with both ACLSM and 2D SIM (Supplementary Figures S1E–G for ACLSM and Supplementary Figures S1I–K for 2D SIM). The mRFP-TUB6-labeled microtubules were only visualized with ACLSM and in this case they were resolved at an average FWHM of 188.044 ± 19.93 nm (mean ± SD; *N* = 35; Supplementary Figure S1D). Likewise, tagRFP-MAP65-2 was resolved by ACLSM at an average FWHM of 176.115 ± 18.08 nm (mean ± SD; *N* = 47; Supplementary Figure S1H) while the respective resolution by 2D SIM was 129.161 ± 14.46 nm (mean ± SD; *N* = 43; Supplementary Figure S1L). Comparison of green with red-labeled structures regardless of the used microscopy platform did not yield any significant differences. The resolution reached for TUA6-GFP and mRFP-TUB6-labeled microtubules was comparable (Supplementary Figures S2A,E). Similarly we did not note any significant differences between tagRFP-MAP65-2 and eGFP-MAP65-2 observed on either ACLSM (Supplementary Figures S2B,E) or 2D SIM (Supplementary Figures S2C,E). By contrast tagRFP-MAP65-2 was considerably better resolved by means of 2D SIM compared to ACLSM (*p* < 0.001) further corroborating the resolution efficiency differences between the two systems (Supplementary Figures 2D,E).

### Detailed View on MAP65-2 Colocalizing With Microtubules

One major point in the analysis of microtubule bundles, is the efficiency with which individual microtubules can be deciphered and co-visualized with other molecules inhabiting the bundle, including bundling proteins such as MAP65-2. To co-visualize microtubules and MAP65-2 and decipher their spatial relations in composite cortical microtubule bundles, we preferentially used ACLSM. 2D SIM was also used, but due to the configuration of the platform, it was possible to acquire images at the two channels sequentially with significant delays compared to ACLSM, where sequential imaging was done considerably faster. Moreover, owing to its detection principle, ACLSM allows the best photon collection even at suboptimal signal-to-noise ratios at approximately the same resolution as 2D SIM (Figures 1, 2; Huff, 2016).

**FIGURE 4:**
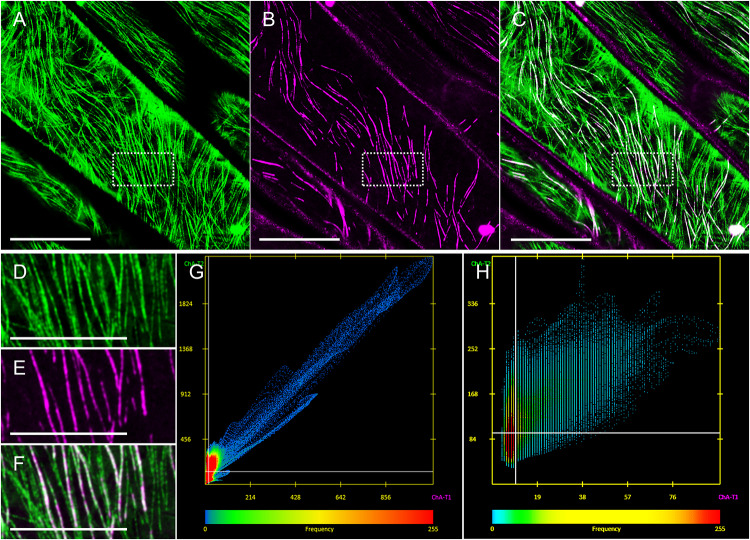
Demonstration and analysis of MAP65-2 colocalization with cortical microtubules. Hypocotyl epidermal cells of stably transformed seedlings expressing both tagRFP-MAP65-2 and TUA6-GFP as visualized by ACLSM (objective 63×/1.40 NA). **(A–C)** Overview of TUA6-GFP labeled microtubules **(A)**, tagRFP-MAP65-2 **(B)**, and their overlay **(C)**. **(D–F)** Magnified views of the boxed area of **(A–C)** showing again TUA6-GFP tagged microtubules **(D)**, tagRFP-MAP65-2 **(E)** and the resulting merged image **(F)**. **(G,H)** Scatterplots showing spatial correlation of green vs. red pixels for the entire field of view shown in C **(G)** or corresponding to the rectangular ROI (boxed area of C; **H**). Scale bars = 10 μm, **(A–C)**; 5 μm, **(D–F)**.

Thus, ACLSM was used for imaging epidermal hypocotyl cells co-expressing eGFP-MAP65-2 with mRFP-TUB6, or tagRFP-MAP65-2 with TUA6-GFP markers. We were able to co-visualize cortical microtubules and MAP65-2, while avoiding bleaching issues that were particularly limiting to tagRFP-MAP65-2 visualization by 2D SIM. To properly address the nature of MAP65-2 colocalization with microtubules, two approaches based on light intensity profiling were used as described previously (Komis et al., 2014). By employing these approaches, we wanted to verify, whether MAP65-2 follows the same trends that were reported for cortical microtubules (Komis et al., 2014). The first trend specifies the composite nature of cortical microtubules, as microtubule bundles containing different number of microtubules can not only be discriminated from each other, but the fluorescence intensity maximum within a microtubule bundle is linearly depending on the number of microtubules incorporated in it. In composite bundles consisting of many microtubules, intensity fluctuations along linear profiles can reflect the number of individual components (Komis et al., 2014). Apart from this goal, we were also interested whether we could observe cases, where the tagRFP-MAP65-2 signal would be close to the signal corresponding to TUA6-GFP-labeled microtubules, yet the signals would not completely overlap, meaning there is MAP65-2 signal outside of the tightly bound microtubules. In quantitative terms, colocalization of tagRFP-MAP65-2 with TUA6-GFP marked microtubules is quite tight as evidenced by colocalization analyses of either the entire cortical microtubule system (Figures 4A–C,G) or selected regions of interest that selectively encompass bundled microtubules (Figures 4D–F,H). Quantitative assessment of tagRFP-MAP65-2 with TUA6-GFP in the full frame shown in Figures 4A–C, showed collinearity of both signals with a Pearson’s coefficient value of 0.964 and a Manders coefficient value of 0.891. In the selected rectangular ROI, the respective Pearson’s coefficient is 0.604 and the Manders coefficient is 0.92.

**FIGURE 5:**
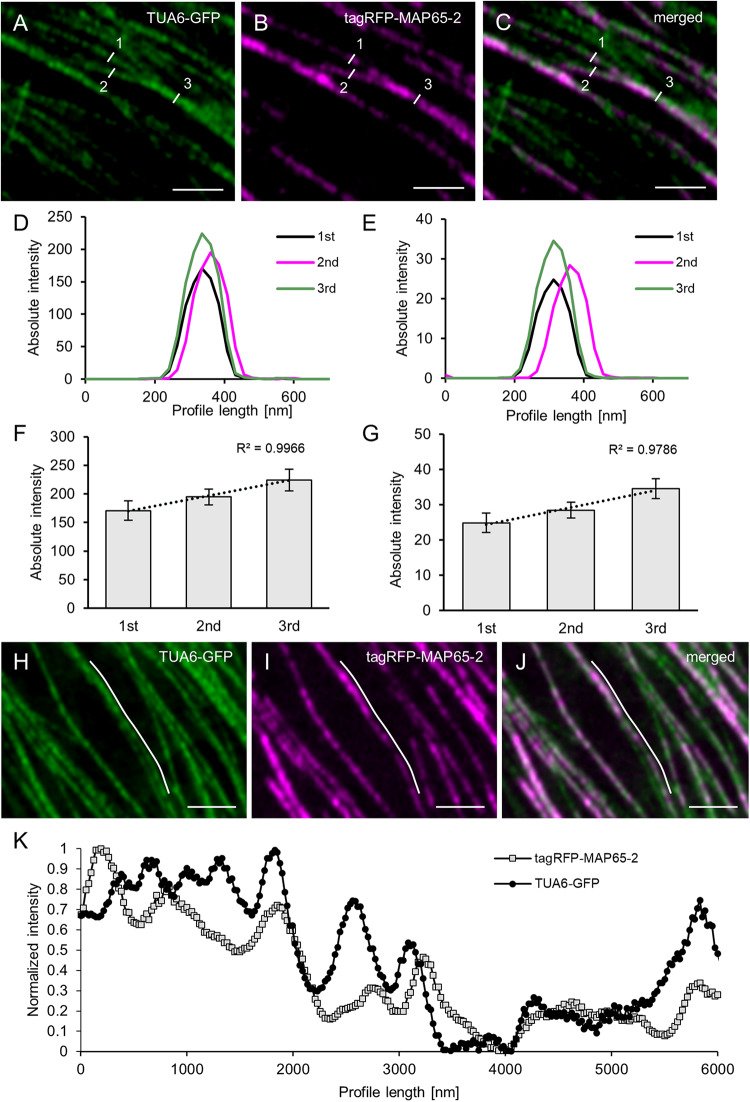
Quantitative analysis of MAP65-2 colocalization with cortical microtubules. Hypocotyl epidermal cells of stably transformed Arabidopsis lines expressing both tagRFP-MAP65-2 and TUA6-GFP were observed in ACLSM (objective 63×/1.40 NA). **(A–C)** Overview of an area with microtubule branching with TUA6-GFP shown in green **(A)**, tagRFP-MAP65-2 in red **(B)** and **(C)** the showing the overlay; measured microtubule bundles are visualized with white lines and labeled with given numbers according to their strength (1 being the weakest and 3 the strongest bundle). Microtubule bundles were quantified by fluorescence intensity profiling and averaged values are shown in **(D)** for TUA6-GFP and **(E)** for tagRFP-MAP65-2. Quantitative evaluation is given in **(F)** for TUA6-GFP and **(G)** for tagRFP-MAP65-2 (mean ± SD; R2, linear correlation coefficient; *N* = 8, 5 technical repetitions). **(H–J)** Overview of a microtubule bundle, TUA6-GFP shown in green **(H)**, tagRFP-MAP65-2 in red **(I)** and **(J)** is a merged picture; white line visualizes longitudinal profile, which is shown in **(K)**, where is demonstrated fluctuation of fluorescence intensities. Scale bars = 2 μm.

To answer the first question regarding the linear increase in signal intensity when microtubule bundles blend together, we searched the images of Arabidopsis line stably expressing both tagRFP-MAP65-2 and TUA6-GFP markers for cases showing events of microtubule bundles branching in two smaller branches (Figures 5A–C). Then, we measured the fluorescence intensity profiles perpendicularly to the original bundle as well as to its two branches. To prevent errors stemming from local deviations in fluorescence intensity maxima of either fluorescent signal, five independent measurements were performed on each measured branch and these data were averaged. Such averaged measurements (*N* = 8; Figures 5D,E), display a clear distinction between the absolute fluorescence intensity maximum of the original bundle, the smaller and the larger branch. Moreover, the linear correlation coefficient between the two signals was inferred from absolute fluorescence intensity maxima of all three microtubule bundles of different complexity (i.e., differing in the microtubule number they accommodate; Figures 5F,G). For both TUA6-GFP-labeled microtubules (Figure 5F) and tagRFP-MAP65-2 (Figure 5G), the high values of the linear correlation coefficient (*R*^2^ = 0.9966, respectively *R*^2^ = 0.9786) confirm the observation, that both microtubules and MAP65-2 accumulate equally during the increase of the bundle size.

Next, using the images of the same Arabidopsis line with two markers tagRFP-MAP65-2 and TUA6-GFP, we focused on areas showing signal intensity fluctuation alongside the decorated microtubules. To examine whether these changes in the signal intensity of TUA6-GFP-labeled microtubules would be mirrored in signal intensity changes of tagRFP-MAP65-2, we draw longitudinal profiles along microtubules. A representative measurement is shown in Figures 5H–K. Due to considerable differences in absolute intensity values between signals corresponding to tagRFP-MAP65-2 and TUA6-GFP, the values from longitudinal profiles were normalized before they were plotted against distance (Figure 5K). Such linear profiles were discontinuous, reflecting the uneven incorporation of TUA6-GFP in the microtubule lattice (Komis et al., 2014) and the similarly uneven binding of MAP65-2 alongside microtubule bundles. Signal intensity fluctuations of tagRFP-MAP65-2 were not coinciding with those of the TUA6-GFP signal. However, the overall changes along both linear profiles showed the same trend of signal intensity increase pending on the increase in bundle complexity, suggesting that the abundance of MAP65-2 at a specific place depends on the composition of a microtubule bundle at that place.

**FIGURE 6:**
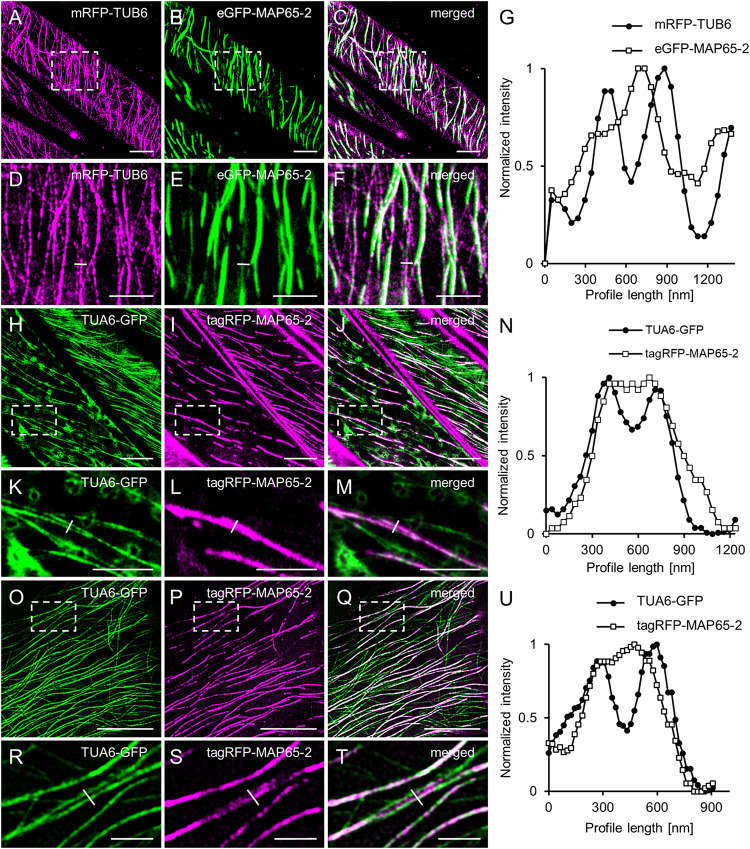
Colocalization of MAP65-2 with microtubules. **(A–C)** ACLSM overview of microtubule bundles in hypocotyl cells coexpressing mRFP-TUB6 **(A)** and eGFP-MAP65-2 **(B)**; merged picture is shown in **(C)**. Boxed area in **(A–C)** is shown in **(D–F)**; **(D)** mRFP-TUB6, **(E)** eGFP-MAP65-2, **(F)** overlay; yellow line in **(D–F)** delineates fluorescence intensity profile, shown in **(G)**. Profile intensity measurement was normalized, plotted against distance and the resulting graph is in **(G)**. **(H–J)** ACLSM co-visualization of microtubule bundles in line coexpressing TUA6-GFP **(H)** and tagRFP-MAP65-2 **(I)**; overlay is displayed in **(J)**. Boxed area in **(H–J)** is shown in **(K–M)**; **(K)** TUA6-GFP, **(L)** tagRFP-MAP65-2 and in **(M)** is overlay; yellow line in **(K–M)** delineates fluorescence intensity profile, shown in **(N)**. After normalization, profile intensity measurement was plotted against distance and the graph is in **(N)**. **(O–Q)** Images of line expressing TUA6-GFP **(O)** and tagRFP-MAP65-2 **(P)** and their colocalization **(Q)**. Boxed area in **(O–Q)** is shown in **(R–T)**; **(R)** TUA6-GFP, **(S)** tagRFP-MAP65-2 and in **(T)** is overlay; yellow line represents a perpendicularly drawn fluorescence intensity profile shown in **(U)**. After normalization, profile intensity measurement was plotted against distance and the graph is in **(U)**. Scale bars = 10 μm **(A–C,H–J,O–Q)**, 5 μm **(D–F,K–M)** or 2 μm **(R–T)**.

Last, during a careful analysis of ACLSM images of Arabidopsis lines carrying eGFP-MAP65-2 and mRFP-TUB6, or tagRFP-MAP65-2 and TUA6-GFP marker couples, respectively, we noted that there were cases, where MAP65-2 was observed to localize both within microtubule bundles and in between two parallel microtubule bundles in the near proximity to each other (Figures 6A–F,H–M). These observations were addressed by normalized intensity profiles drawn perpendicularly to composite microtubule bundles (Figures 6G,N). The graphs confirmed the visual observation and further demonstrated association of MAP65-2 with microtubule bundles and its localization between these bundles by showing that peak intensities of TUA6-GFP and tagRFP-MAP65-2 are offset (Figures 6G,N). To strengthen our observation and to dismiss the possibility of noting this information due to poor resolution, we decided to check whether similar situation can be found in images with higher resolution. For this, we used SIM with a 100×/1.46 NA, oil-immersion objective. Again, the same observation was confirmed (Figures 6O–T) and proved in normalized fluorescence intensity profile plotted against distance (Figure 6U) showing offset position of peak intensities of TUA6-GFP and tagRFP-MAP65-2.

### Dynamics of MAP65-2 Localization

**FIGURE 7:**
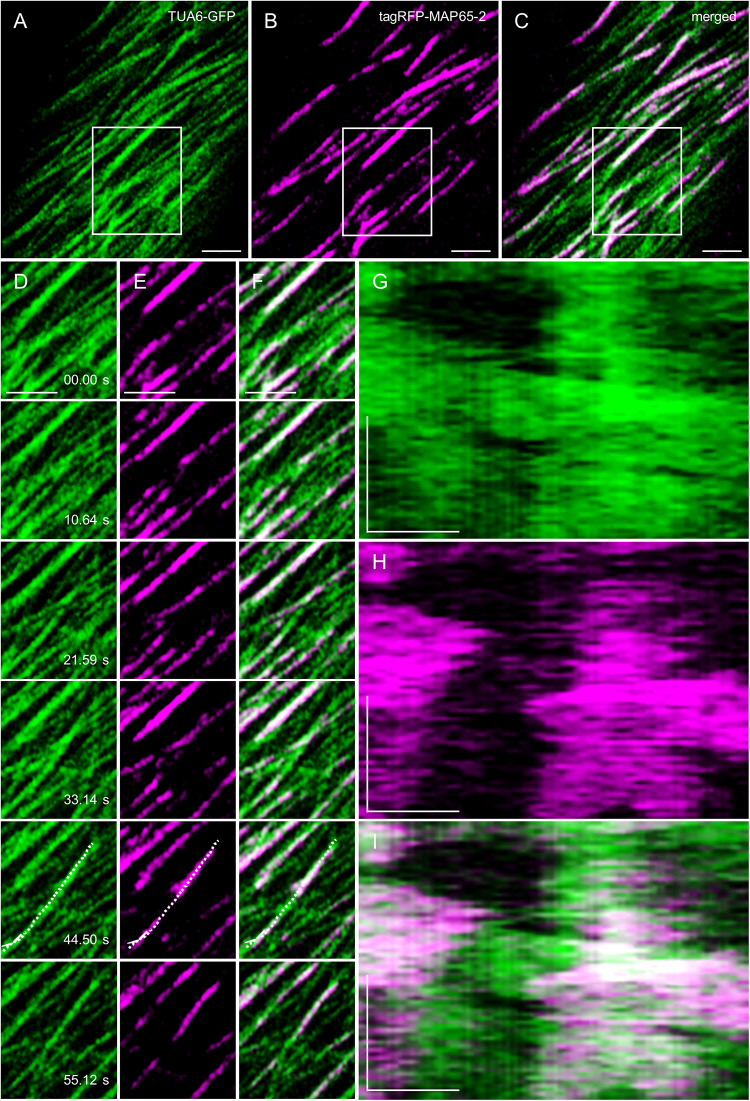
MAP65-2 and microtubule dynamics visualized by ACLSM (see also Supplementary Video S1). **(A–C)** Is an overview of a hypocotyl epidermal cell of stably transformed line expressing both TUA6-GFP **(A)** and tagRFP-MAP65-2 **(B)** observed by ACLSM (objective 63×/1.4 NA); in **(C)** is merged picture. Area in boxes is shown in stills **(D–F)**, within which is the region of interest where kymographs were generated **(G–I)**. Stills in **(D)** show TUA6-GFP, in **(E)** show tagRFP-MAP65-2 and in **(F)** are stills from merged picture. In **(G)**, kymograph is shown for TUA6-GFP, in **(H)** for tagRFP-MAP65-2 and in **(I)** for merged picture. Scale bars = 2 μm **(A–F)**, 1 μm **(G–I)**. Time bars = 1 min **(G–I)**.

Due to the ever-changing nature of cortical microtubules, MAP65-2, as a protein associated with microtubules, is expected to follow microtubule dynamics (e.g., Lucas et al., 2011). Concomitant recordings tracking the dynamics of both eGFP-MAP65-2 and mRFP-TUB6 were done using the ACLSM at frame rates of ca. 0.67 fps (time interval of 1.5 s). The eGFP-MAP65-2 follows microtubule labeling at areas of potential antiparallel microtubule overlaps (Figures 7A–F and Supplementary Video S1), whereas the length fluctuations of eGFP-MAP65-2 and mRFP-TUB6 closely follow each other (Figures 7G–I).

For more detailed analysis, we restricted time lapsed imaging to eGFP-MAP65-2. To address this issue, we employed 2D SIM and TIRF-SIM to survey eGFP-MAP65-2 dynamics at different temporal resolutions. In both cases, it was possible to make recordings of durations ranging between 10 and 30 min, but with markedly different acquisition frame rates. With 2D SIM, images were acquired at frame rates between 0.22 fps to 0.4 fps (time intervals from 2.5 s to 4.5 s) while the TIRF-SIM recordings were done at frame rates between 1.33 fps to 10 fps (time intervals from 750 to 100 ms).

Since MAP65-2 is preferentially crosslinking antiparallel microtubules, the end-wise dynamics of eGFP-MAP65-2 do not follow the classical view of microtubule dynamics *per se*. MAP65-2 is closely tracking antiparallel microtubule plus ends and it only persists as long as the overlap between the microtubules does (Lucas et al., 2011). This was previously shown by simultaneous tracking of mCherry-MAP65-2 and GFP-TUA6 (Lucas et al., 2011), proving that end-wise microtubule dynamics are closely followed by approximate changes in the length of the MAP65-2 signal.

**FIGURE 8:**
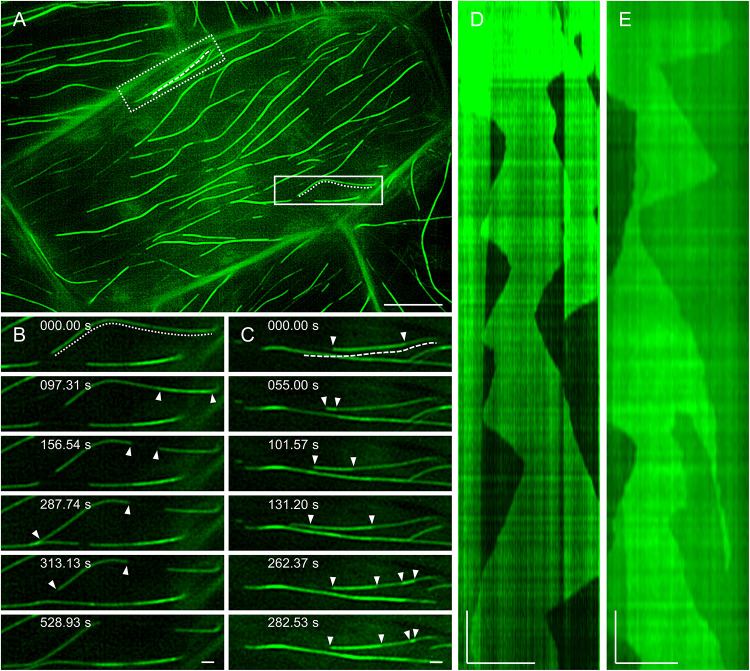
MAP65-2 dynamics (see also Supplementary Video S2). **(A)** An overview of a hypocotyl epidermal cell of stably transformed line expressing eGFP-MAP65-2 observed by 2D-SIM (objective 63×/1.40 NA). In full-line box is area, which is magnified in stills in **(B)** and the dotted line marks microtubule bundle used for generating kymograph **(D)**; while in dotted-line box is area depicted in stills in **(C)**, with dashed line next to microtubule bundle used for generating kymograph in **(E)**. Stills in **(B)** show growth of an individual microtubule bundle as is demonstrated on a kymograph **(D)**. Complex dynamics within composite microtubule bundle are demonstrated in stills **(C)** and a corresponding kymograph **(E)**; white arrowheads point to microtubule ends. Scale bars = 10 μm **(A)**, or 1 μm **(B–E)**. Time bars = 1 min **(D,E)**.

In agreement with previous observations (van Damme et al., 2004; Lucas et al., 2011), the dynamic behavior of MAP65-2 is similar to that of cortical microtubules. With 2D SIM, MAP65-2 decorating overlaps of antiparallel cortical microtubules displays periods of growth (extension) followed up with fast shrinkage (retraction; Figures 8A,B and Supplementary Video S2), mimicking the dynamic instability of plus ends of cortical microtubules. To quantify this behavior, kymographs were generated from the original images and parameters describing MAP65-2 dynamics were extrapolated from these kymographs (Figure 8D). In this case, the growth and shrinkage rates were 6.87 ± 2.72 μm min^–1^ (*N* = 57) and 21.35 ± 6.38 μm min^–1^ (*N* = 43), respectively. Moreover, frequencies of rescue and catastrophe events were 0.0107 and 0.0446, respectively.

2D SIM allowed to pursue dynamics of eGFP-MAP65-2 in more complex bundles, presumably accommodating more than two microtubules (Figures 8A,C). This is deemed by the higher fluorescence intensity and it is reflected in the resulting kymographs exhibiting areas of variable intensity above the background fluorescence (Figure 8E). Again, inferring from kymographs (Figure 8E), the growth and the shrinkage rates were 6.32 ± 2.00 μm min^–1^ (*N* = 42) and 17.08 ± 4.84 μm min^–1^ (*N* = 30), respectively. The frequencies of rescue and catastrophe events were 0.0114 and 0.0404, respectively.

**FIGURE 9:**
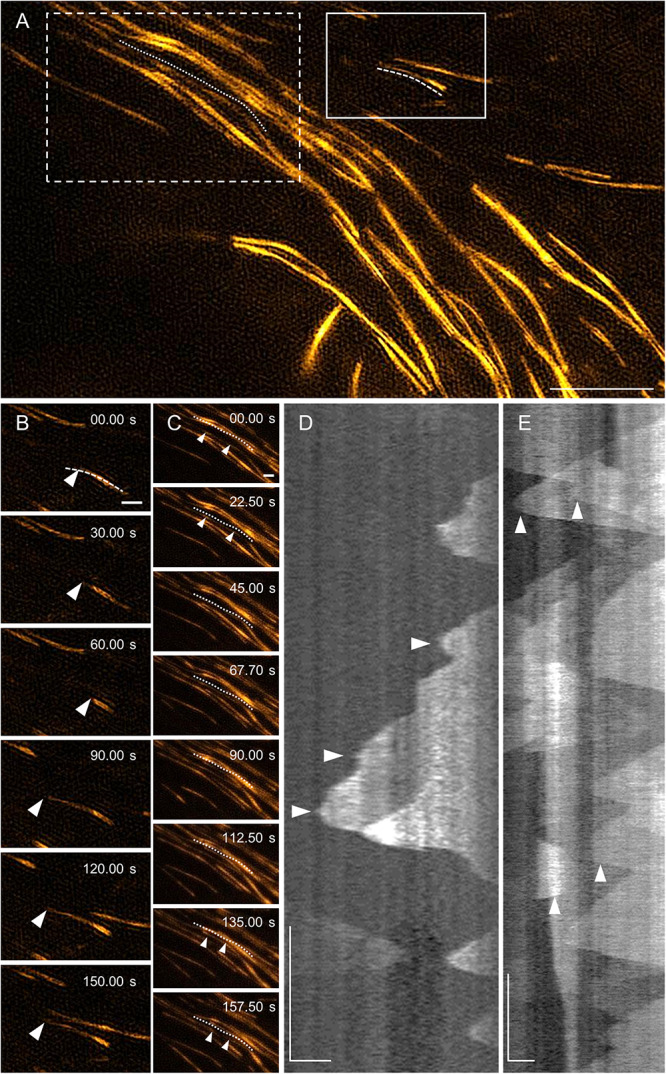
MAP65-2 dynamics visualized by TIRF-SIM (see also Supplementary Video S3). **(A)** An overview of a hypocotyl epidermal cell of stably transformed line expressing eGFP-MAP65-2 observed by TIRF-SIM (objective 100×/1.49 NA). In full-line box is area, which is magnified and shown in stills in **(B)**, with dashed line marking microtubule bundle used for generating kymograph in **(D)**, while in dotted-line box is area depicted in stills in **(C)** and the dotted line is next to microtubule bundle, from which kymograph of **(E)** was generated. Stills in **(B)** show growth of a plus end of an individual microtubule bundle as demonstrated on a kymograph **(D)**. A complex dynamics within composite microtubule bundle is demonstrated in stills **(C)** and a corresponding kymograph **(E)**; white arrowheads point to microtubule ends. Scale bars = 5 μm **(A)**, or 1 μm **(B–E)**. Time bars = 1 min **(D,E)**.

Similar analysis was done using TIRF-SIM (Figures 9A–C and Supplementary Video S3), after the subsequent generation of kymographs (Figures 9D,E). Growth and shrinkage rates deduced from TIRF-SIM images were calculated as 5.04 ± 1.30 μm min^–1^ (*N* = 53) and 18.27 ± 5.04 μm min^–1^ (*N* = 36), respectively, which is comparable to the results from SIM. However, the frequencies of rescue and catastrophe events were in both cases higher compared to 2D SIM (0.0381 and 0.0864, respectively), probably due to the higher spatial and temporal resolution, which enabled more detailed measurement of dynamic changes.

**FIGURE 10:**
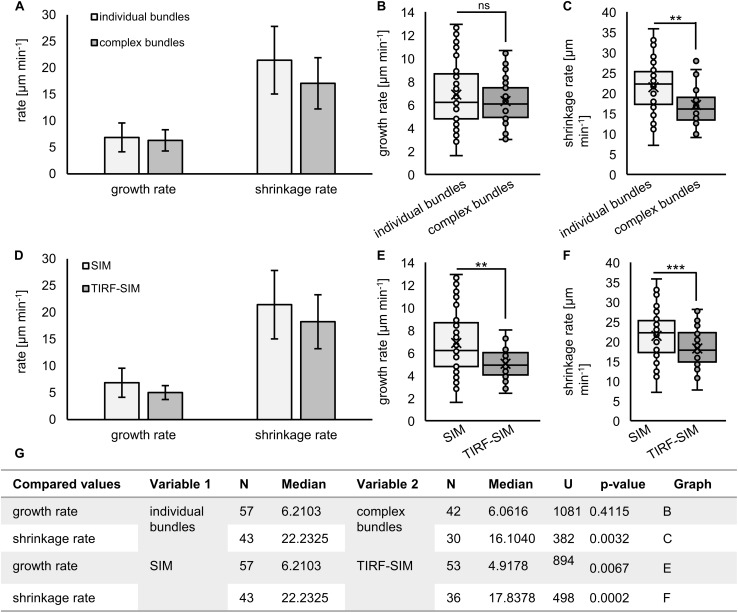
Quantification of MAP65-2 dynamics. Kymographs were generated from videos of stably transformed line expressing eGFP-MAP65-2 made by either 2D SIM (objective 63×/1.40 NA) or TIRF-SIM (objective 100×/1.49 NA). The kymographs were measured and growth and shrinkage rates were calculated (as described in section “Materials and Methods”). **(A–C)** Comparison of growth **(A,B)** and shrinkage **(A,C)** rates for individual and complex bundles observed on 2D SIM. **(D–F)** Comparison of growth **(D,E)** and shrinkage **(D,F)** rates for bundles imaged by either 2D SIM or TIRF-SIM. In **(A,D)**, means and standard deviations are shown. In **(B,C,E,F)**, results from statistical analysis are shown, for which was used Mann–Whitney *U* test (*** significant at *p* 0.001, ** significant at *p* 0.01, ns, not statistically significant) and the results of statistical analysis are in **(G)**, where U is the result of Mann–Whitney test. Description of box plot: average is presented by ×, median by the middle line, 1st quartile by the bottom line, 3rd quartile by the top line; the whiskers lie within the 1.5 × interquartile range (defined from the 1st to the 3rd quartile), outliers are presented by points.

Growth rates within individual bundles are similar to those calculated from more complex bundles but shrinkage is slower (Figures 10A–C,G). When comparing the two acquisition methods, both growth and shrinkage rates inferred by TIRF-SIM, were significantly slower than those obtained by 2D SIM (*p* = 0.0067 for growth; Figures 10D,E,G and *p* = 0.0002 for shrinkage; Figures 10D,F,G). This is probably owing to the big difference between the two systems in terms of sampling frame rates. Since TIRF-SIM has essentially the same resolution potential like 2D SIM, it is probably collecting more frames without detectable length changes compared to 2D SIM.

### Single Molecule PALM Localization of mEos3.2-MAP65-2

**FIGURE 11:**
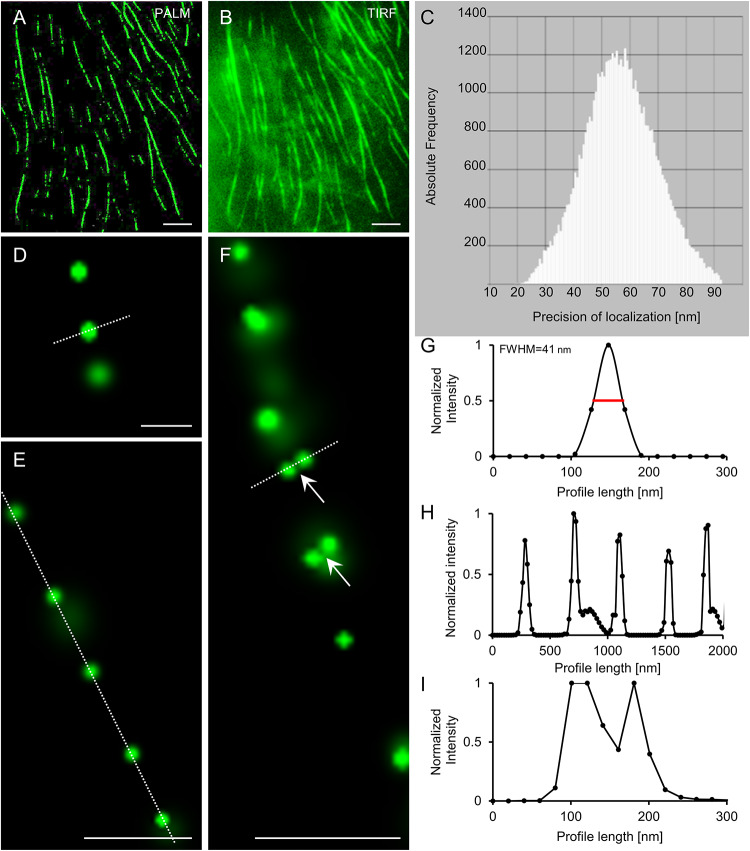
PALM localization of mEos3.2-MAP65-2. **(A,B)** Overview of Arabidopsis hypocotyl epidermal cell expressing mEos3.2-MAP65-2 at the end of an acquisition time series following single molecule localization by PALM **(A)** or TIRF imaging **(B)**. **(C)** Histogram of photon detection frequencies plotted against precision of localization. **(D–F)** Details from PALM imaging in **(D)** are single mEos3.2-MAP65-2 molecule; **(E)** shows a linear array of single mEos3.2-MAP65-2 molecules; and in **(F)** is a linear array of mostly mEos3.2-MAP65-2 molecules with two pairs of spots most likely corresponding to mEos 3.2-MAP65-2 dimers. **(G)** Normalized intensity profiling of the spot delineated with the dotted white line in **(D)**, showing a full-width at half maximum (FWHM) of ca. 41 nm. **(H)** Normalized intensity profiling corresponding to the dotted white line in **(E)**, showing the periodic distribution of mEos3.2-MAP65-2 along a microtubule bundle. **(I)** Normalized intensity profiling along the dotted white line of **(F)**. Scale bars = 5 μm **(A,B)**; 0.2 μm **(D)**; 0.5 μm **(E,F)**.

For visualization of the mEos3.2-MAP65-2 reporter we followed previously published settings to induce photoconversion and collect photons (Hoogendoorn et al., 2014; Hosy et al., 2015). The mEos3.2-based reporter of MAP65-2 reacted reasonably well albeit slowly during photoconversion experiments and image reconstruction after the acquisition of time series consisting of ca. 8,000–12,000 frames. Following the photoconversion of mEos3.2, PALM reconstruction resulted in the localization of well discernible single molecules compared to the respective TIRF image, where the signal was roughly continuous (Figures 11A,B). Individual localization events were recorded by variable photon numbers (Figure 11C) resulting to a precision ranging between 10 and 80 nm (38 nm ± 19 nm; mean ± SD; Figures 11D,G). In areas presumably corresponding to microtubule bundles, localization events exhibited an arrayed order by comparison to the continuous localization via TIRF (Figures 11A,B,E), which was further proven upon intensity profile quantification (Figure 11H). In most cases, localization resulted in the documentation of individual spot-like structures, possibly corresponding to individual mEos3.2-MAP65-2 molecules and not dimers (Figures 11D,E). Occasionally we documented spot duplets, i.e., spots that are very closely positioned (Figure 11F). Normalized fluorescence intensity profiling and averaging of the Rayleigh distances (at ca. 67% of the peak intensity) proved a resolution of 30.53 ± 5.28 (mean ± SD; *N* = 17; Figure 11I). Given that the physical length of the closely related MAP65-1 dimer is ca. 25 nm as judged from transmission electron micrographs of *in vitro* reconstituted microtubule bundles after negative staining (Tulin et al., 2012) it is likely that these localization events revealed by optical nanoscopy correspond to true MAP65-2 dimers.

**FIGURE 12:**
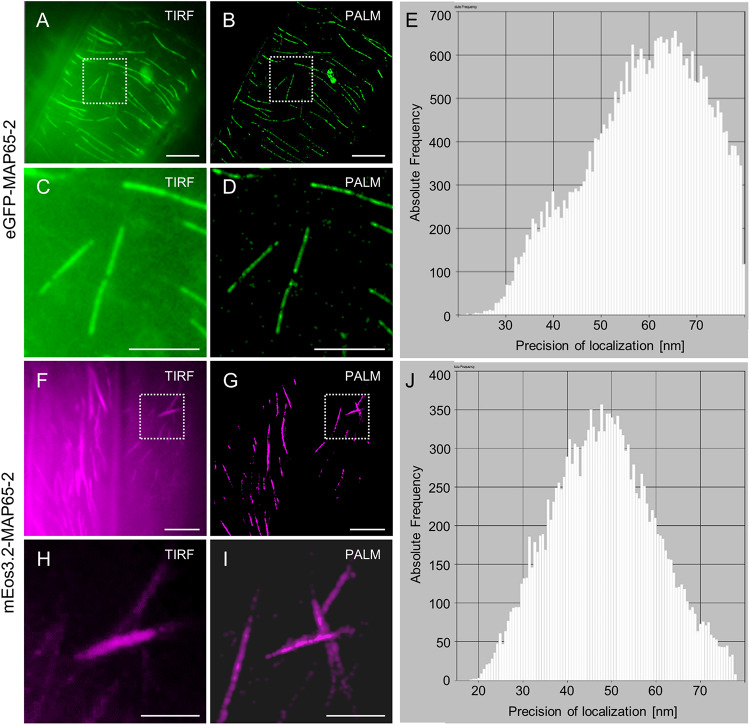
Comparison of single molecule localization microscopy (SMLM) between eGFP-MAP65-2 stochastic optical fluctuations and mEos3.2-MAP65-2 photoconversion. **(A,B)** Comparative overviews of a hypocotyl cell expressing eGFP-MAP65-2 visualized after TIRF illumination **(A)** or following PALM reconstruction **(B)**. **(C,D)** Detailed comparisons of a magnified view of the boxed area in **(A,B)** under TIRF illumination **(C)** or PALM reconstruction **(D)**. **(E)** Histogram of precision of localization plotted against photon frequency. **(F,G)** Comparative overviews of a hypocotyl cell expressing mEos3.2-MAP65-2 by means of TIRF **(F)** or after PALM reconstruction **(G)**. **(H,I)** Detailed view of the boxed areas in **(F,G)** after TIRF acquisition **(H)** or PALM reconstruction **(I)**. **(J)** Histogram of precision of localization plotted against photon frequency. Scale bars = 10 μm **(A,B)**; 5 μm **(C,D,F,G)**; 2 μm **(H,I)**.

Similar results were obtained when optical fluctuations of eGFP-MAP65-2 were recorded using TIRF illumination, a high laser power input at the sample and simultaneous illumination with the 405 nm laser line of Elyra PS.1 (Figures 12A–E). The major difference when comparing with mEos3.2 (Figures 12F–J), was that with eGFP-MAP65-2 we achieved much higher photon numbers per position, very rapidly, minimizing the time necessary to yield similar localization precision (Figures 12E,J).

## Discussion

The plant cytoskeleton consists of fine molecular structures, which cannot be properly characterized by means of light microscopy with the Abbe’s limitation of 200 nm due to diffraction. Instead, transmission electron microscopy was used to describe microtubules and their complex nature of interactions with a pleiade of MAPs (e.g., Ledbetter and Porter, 1963; Chan et al., 1999). Despite its superior resolution and its irreplaceable role for examining supramolecular structures, protein colocalization and interaction (Celler et al., 2016), a great disadvantage of electron microscopy is visualization of only fixed and artificially contrasted samples. On the other hand, intracellular structures show inherent dynamic redistribution during the time of observation and cortical microtubules of plant cells represent such an example. Cortical microtubules in *A. thaliana* exhibit rapid length fluctuations preferably at their plus ends, although occasionally they may oscillate via their minus end in a course of hybrid treadmilling (Shaw et al., 2003; Komis et al., 2014). Moreover they tend to accommodate into higher order assemblies incorporating more microtubules which are physically crosslinked by a number of appropriate microtubule proteins including members of the MAP65 (reviewed in Hamada, 2014) and MAP70 (Korolev et al., 2005, 2007; Pesquet et al., 2010) families. These proteins form subresolution crossbridges that are impossible to discern from microtubules with standard diffraction-limited fluorescence microscopy approaches.

To make ends meet, a plethora of superresolution techniques, which stem from light microscopy and circumvent the Abbe’s limit, were developed with the premise to survey and temporally follow intracellular organization at the nanoscale. Among them, several were described as especially suitable for characterizing both organization and dynamics of microtubules and MAPs, namely SIM, PALM and STORM (Komis et al., 2015, 2018; Schubert, 2017). The SIM method proved to describe microtubule organization and dynamics with high resolution (Komis et al., 2014), reaching as low as nearly a half of Abbe’s diffraction limit. 2D SIM as applied by all existing commercial platforms offers acceptable acquisition frame rates regarding microtubule dynamics (e.g., Shaw et al., 2003; Buschmann et al., 2010; Lucas et al., 2011; Komis et al., 2014, 2015).

With the implementation of TIRF and a unique strategy to generate and move the patterned light, this technique is capable of capturing time-lapsed images with high temporal resolution (Vizcay-Barrena et al., 2011). On the other hand, SIM relies on computer-assisted image reconstruction, which makes SIM prone to a variety of artifacts (see comments in Komis et al., 2015; Demmerle et al., 2017). Nevertheless, due to the aforementioned advantages and well-established protocols of image acquisition and subsequent reconstruction (Komis et al., 2014, 2015), SIM proved to be a valuable tool for determining cytoskeletal dynamics.

To compare SIM, TIRF-SIM and ACLSM regarding their abilities to resolve microtubular structures, we have used these techniques to visualize cortical microtubules in hypocotyl epidermal cells of *A. thaliana*, an approach generally used when describing interphase microtubular array (Kawamura et al., 2006; Pastuglia et al., 2006). The *A. thaliana* lines carrying fluorescently labeled tubulin (TUA6-GFP or mRFP-TUB6) or fluorescent protein fusions with MAP65-2 were used in this study. Previously, the TUA6-GFP marker has been shown not to interfere with microtubule function and dynamics as marked from the lack of phenotypes of the transformants (Shaw et al., 2003).

As for the fluorescently labeled MAP65-2 marker, it was designed in a way to ensure proper function and localization of the resulting chimeric protein. Thus, it is expressed under native promoter while the fluorescent protein is fused with N-terminal sequence of MAP65-2, which is not responsible for binding to microtubules. All seedlings used herein are T3 generation and were all chosen based on fluorescence intensity uniformity and the absence of phenotype when compared to same age untransformed Col-0 seedlings. Accordingly, we did not observe differences in the dynamics of MAP65-2 nor to its patterns of localization in the cortical array compared to previously published work (Lucas et al., 2011), suggesting the functionality of all MAP65-2 fusion proteins used herein (eGFP-MAP65-2, tagRFP-MAP65-2, and mEos3.2-MAP65-2).

Images acquired from these two lines were quantitatively evaluated, using FWHM as a value of the resolution capacity of each respective microscope. By using the same objective, (63×/1.40 NA), the SIM platform outperformed ACLSM. Accordingly, when using objective with higher magnification and NA (100×/1.49 NA), the resolution reached by TIRF-SIM further increased. Our results are in agreement with previously published data for 2D SIM (Komis et al., 2014). However, both ACLSM and SIM were shown to be capable of nearly linear titration of bundled microtubule numbers by measuring fluorescence intensity.

Both 2D SIM and TIRF-SIM, allowed us to track end-wise dynamics of MAP65-2 length fluctuations in either microtubule pairs, or more complex microtubule assemblies. Our results show that the dynamics of MAP65-2 length changes are tightly coupled to end-wise length fluctuations of antiparallel microtubules as described before (Lucas et al., 2011). Within bundles, speckles of MAP65-2 remain immobile, further proving the end-wise restriction of MAP65-2 dynamics and the stability of intrabundle crosslinks. Dynamicity values of MAP65-2 excursions of eGFP-MAP65-2 expressors were considerably slower when analyzed with TIRF-SIM compared to 2D SIM. In particular, both extensions and retractions of the MAP65-2 signal as imprinted in the respective kymographs occurred at a slower pace compared to the respective 2D SIM acquisitions. This observation raises the issue of temporal sampling taking into account the resolution of the system used. Tracking of MAP65-2 dynamics is spatially limited by the system. If such changes occur at smaller lengths than what the system is able to resolve, then they will not be recorded as such. Therefore length fluctuations within the 4.5 s interval of the 2D SIM are expected to be bigger than those recorded with the 100–750 ms interval of the TIRF-SIM platform and also account for differences in transition frequencies (designated as catastrophes and rescues to follow the microtubule nomenclature).

The benefit of both systems, however, was the possibility to contrast intensity differences of variable MAP65-2 intensities and discriminate dynamics between individual components as was previously done with uniformly labeled microtubules (Komis et al., 2014). In the future, fast SIM platforms can be used to survey the dynamic nature of microtubule complexity in bundled systems by simultaneously addressing the distribution of more than one component at the time.

In this respect and owing to the default setup of the 2D SIM microscope used herein, it was not possible to acquire simultaneously signals from both tagged MAP65-2 and tagged tubulin. This constitutes a particular problem, since both microtubule length and MAP65-2 dynamics show considerable changes within the time frame of 2D SIM acquisitions followed herein and would not allow the faithful tracking of both components at the sequential mode of acquisition used. For this reason, ACLSM imaging was employed instead, since quantitative analysis of the imaging output showed that it still exhibited a resolution below Abbe’s limit although significantly lower than that achieved by 2D SIM. Notwithstanding, with the introduction of Airyscan, an improved detector designed for ACLSM, the scanning time was dramatically shortened, while the main advantage of CLSM for live cell imaging, namely low phototoxicity, remained. So far, a direct comparison between SIM and ACLSM has not been provided.

A clear benefit of 2D-SIM and TIRF-SIM is the highly contrasted discrimination of intrabundle microtubules within the diffraction limitations of both modalities. Quite surprisingly, the recording of intrabundle dynamics consistently yielded statistically significant lower shrinkage rates compared to individual microtubules. This is quite surprising given that at least *in vitro*, MAP65 proteins did not affect microtubule assembly and disassembly rates (Stoppin-Mellet et al., 2013) but rather the extent and duration of growth and shrinkage. Moreover, they might be also implicated in the decrease of catastrophe frequencies, since they are competing katanin activity (Stoppin-Mellet et al., 2013; Burkart and Dixit, 2019), leading to sustainable intrabundle microtubule elongation. By contrast, *in vivo* observations herein, show striking similarity in both catastrophe and rescue frequencies when comparing intrabundle with individual microtubules. Additionally previous *in vivo* observations, deduced reduced shrinkage rates of intrabundle microtubules as found here (van Damme et al., 2004; Lucas et al., 2011). In contrast to *in vitro* observations, intrabundle microtubule dynamics *in vivo* occur in the context of competitive or synergistic interactions from more protein species than those addressed in *in vitro* assays. Notable interacting partners of bundling proteins such as the fission yeast homolog of MAP65s, Ase1p, are kinesin motors and the plus-end binding protein CLASP (Bratman and Chang, 2007; Janson et al., 2007). This finding will, be further pursued in the near future and it represents a merit of the implementation of live superresolution imaging for *in vivo* observations.

To overcome the dual localization caveat, ACLSM was used in a superresolution fast mode to simultaneously track more than one channel at a time (Huff, 2016; Korobchevskaya et al., 2017). Despite the high resolution capacity of 2D SIM and TIRF-SIM, these platforms exhibit significant phototoxicity resulting in progressive photobleaching of fluorophores and cell damage after the time frames of observation used herein. Although the phototoxicity was not a particular problem for imaging lines expressing GFP-labeled proteins (owing to lower laser inputs and camera exposure times necessary for documentation), it represented a serious predicament for live imaging of mRFP- and tagRFP-labeled proteins. The output of ACLSM is at large comparable in terms of resolution with the output of 2D SIM, excluding the time restrictions of the latter. Taking into account the above advantages and restrictions of the different SIM modules and ACLSM, we used the latter to address aspects of MAP65-2 localization, relationship with microtubules within cortical bundles of variable complexity. Our results show that MAP65-2 either as an eGFP- or as a tagRFP-fusion, partially colocalizes with microtubule overlaps after tracking of microtubules with either TUA6-GFP, or mRFP-TUB6. The labeling of MAP65-2 in all cases was discontinuous without conspicuously following the similar speckled distribution of tagged tubulin. This means that MAP65-2 (as probably happens with other members of the MAP65 family) does not show a binding prevalence to a specific tubulin isoform. Moreover, the uneven distribution of MAP65-2 signifies the fact that microtubule bundles may stochastically recruit different MAP65 proteins during their assembly, including the wild type untagged MAP65-2 protein, which is also expected to be expressed in our transformants. To this extent, it would be of interest in a future study to address the spatial relationships between MAP65-1, MAP65-2, and MAP65-5 which have been shown to coexist in the cortical microtubule array (van Damme et al., 2004; Lucas et al., 2011; Lucas and Shaw, 2012) and to delineate their dynamics.

Diffraction limits of widefield imaging have been surpassed either by physically restricting emission to subdiffraction sizes by means of stimulated emission depletion microscopy (reviewed in Sahl et al., 2017), or by specifying the localization of single fluorophores at nanometer precision. The latter approach encompasses a high number of comparable methods all of which take into account non-linear responses of the fluorophore to excitation conditions. So-called single molecule localization microscopy (SMLM) methods which emerged on this principle rely on either fluorophores switching between on and off states, converting between two different emission peaks, or in intensity fluctuations of single emitters under special excitation conditions.

PALM is based on the first principle and employs photoswitchable, photoactivatable or photoconvertible protein tags (such as PA-GFP, Dronpa, or mEos3.2 as used herein; reviewed in Shcherbakova et al., 2014) which are shifting between two temporally distinct emission states upon appropriate illumination. Stochastic optical reconstruction (STORM) is also based on the on/off transitions of special fluorophores such as AlexaFluor 647 or Atto488 under redox conditions and high irradiance with the excitation wavelength promoting the blinking of individual fluorophores during time-resolved acquisitions only when the sample is embedded in the presence of reducing agents such as β-mercatoethanol or mercaptoethylamine (reviewed in Li and Vaughan, 2018; Jradi and Lavis, 2019).

In plant research, PALM and STORM applications of SMLM are still limited but promising. They have been applied for counting molecules of active and inactive RNA polymerase II in interphase nuclei of Arabidopsis (Schubert and Weisshart, 2015), or to elucidate the organization of perinuclear actin in living tobacco cells (Durst et al., 2014). Together with other studies, which elaborated applications of direct STORM in fixed plant samples interrogating cortical microtubule structure (Dong et al., 2015) or cellulose microfibril arrangement in plant cell walls (Liesche et al., 2013), and PALM combined with single particle tracking of diffusing membrane proteins (Hosy et al., 2015) makes SMLM methods tractable approaches for quantitatively interrogating plant intracellular and extracellular architecture at all spatial dimensions and in time.

Owing to the composite and crowded nature of cortical microtubule bundles and their molecular complement, SMLM methods for visualization of individual components presents ideal means to characterize their molecular composition. To this extend, we followed the PALM principle in order to address the localization of single molecules of mEos3.2-MAP65-2 fusions. The localization process required lengthy time acquisitions, in order to yield photon frequencies necessary for subdiffraction localization precision. Taking into account the dynamic nature of MAP65-2 at the fluctuating ends of overlapping microtubules, the results presented herein are only valid for localizations within the overlap and away from the microtubule tips.

PALM imaging within immotile regions of microtubule bundles showed the definite localization of MAP65-2 crossbridges with outstanding resolution and, most importantly, the discontinuous manner of MAP65-2 decoration as roughly shown by 2D SIM and ACLSM. In our work we could not observe a global periodicity of localized mEos3.2-MAP65-2, compared to TEM observations of reconstituted microtubule bundles. We believe that there might be three reasons for this: (a) not all mEos3.2-MAP65-2 were localized during the acquisition time series, (b) mEos3.2-MAP65-2 is buffered by endogenous MAP65-2 and (c) unlabeled crossbridges may be formed by other members of the MAP65 family such as MAP65-1 (which has overlapping localization with MAP65-2; Lucas et al., 2011) or MAP65-5 (van Damme et al., 2004).

Similar results in terms of precision of localization were obtained when visualizing eGFP-MAP65-2 under conditions of high irradiance with the 488 nm laser and concomitant excitation with the 405 nm line. In terms of efficiency, the photon frequencies necessary for a certain precision of localization were much higher in the case of mEos3.2 photoconverters compared to eGFP molecules with fluctuating intensity. However, in the latter case, precision of localization was roughly similar to that achieved by mEos3.2 photoconversion in much narrower time frames.

Based on our preliminary results, the sparse detection of spot duplets by PALM and the separation distance between individual spot of the duplets, we postulate that these might represent resolved dimers of MAP65-2. The two mEos3.2 moieties of a presumable mEos3.2-MAP65-2 dimer can be separated at a physical distance within the resolution potential of PALM. The robust detection of dimers vs. monomers requires a fluorescent tag with low duty cycle (i.e., the ratio of time spent in the on state vs. the time spent in the off state) and high contrast between the two states in order to avoid overlaps during the detection. Apparently, mEos3.2 fulfils such prerequisites (duty cycle of mEos3.2 is 3 × 10^−6^ and contrast is 200; Li and Vaughan, 2018) and work is under way to map the oligomerization state of MAP65-2. In this direction, it will be necessary to address the composition of microtubular bundles by multichannel localization of differentially tagged MAP65 isoforms. Further studies awaiting next generation microscopic technologies will help to decipher the topology of MAP65 crossbridging of microtubules in diverse plant microtubule arrays that rely on MAP65-mediated bundling, such as biased parallel cortical microtubule arrays, but especially robust 3-D structures, namely preprophase band, mitotic spindle and phragmoplast. More importantly, the recent release on new SIM platforms allowing faster and more light-efficient imaging at multiple channels (commented in Vavrdová et al., 2019b), will help to dynamically address the process of bundle formation in the cortical cytoplasm in plants co-expressing microtubule and various isoform-specific MAP65 markers.

The possibilities of multichannel PALM will facilitate colocalization studies of either microtubules and single MAP65 isoforms, or the spatial relation between different MAP65 isoforms that may redundantly localize within the same microtubule bundle. TIRF-SIM as well as the recent implementation of lattice SIM in commercially available system (see Komis et al., 2018) allow the high speed tracking of microtubule dynamics and particularly the latter modality is constructed to permit multicolor imaging at very high frame rates. Unfortunately lattice SIM is limited to the same resolution range as standard SIM used herein, so it will not able to discriminate between microtubules and MAP65 crossbridges. However, the lattice SIM system may be suitable for the volumetric dynamic colocalization of microtubules and different MAP65 isoforms a task currently impossible for most SIM modalities existing today. The intrabundle dynamics of individual MAP65 molecules may be addressed by single particle tracking PALM, which is a goal for the immediate future.

Another limitation of the methods presented herein, is related to the duration of observations. As mentioned before, many events leading to microtubule reordering occur over time and their successful documentation requires excitation light inputs that will not harm the sample. The SIM (and the TIRF-SIM) modalities used herein are likely prone to phototoxicity-related artifacts and cell damage and this is why imaging sequences were time limited. Although this is beyond the scope of the present study, it will be a future goal to investigate the potential of Airyscan CLSM to this respect since by principle of detection, it does not require a high laser input to achieve superresolution output.

## Data Availability Statement

All material integral to the present study, will become available upon reasonable request from the corresponding author (GK, georgios.komis@upol.cz).

## Author Contributions

TV, PK, and PI were involved in generation of all material used herein (cloning, transformations, crosses). TV, PK, PI, PF, and GK were involved in selection of transgenic plants used for propagation and imaging. TV, RŠ, and PF managed plant handling. TV, MO, OŠ, PF, RŠ, and GK were involved in image acquisition and carried out all post-acquisition image processing regarding 2D SIM, ACLSM, TIRF-SIM, and PALM. TV and GK conducted all post-acquisition image analysis. TV and GK drafted the manuscript and figures with input and editing by PK, OŠ, MO, and JŠ. GK and JŠ provided the funding resources. JŠ provided infrastructure.

## Conflict of Interest

The authors declare that the research was conducted in the absence of any commercial or financial relationships that could be construed as a potential conflict of interest.

## Footnotes

1 https://www.embl.de//eamnet/html/body_kymograph.html

## Supplementary Material

FIGURE S1 Resolving details of cortical microtubules with mRFP-TUB6 marker or MAP65-2 decoration of cortical microtubules with tagRFP-MAP65-2. The signal of mRFP-TUB6 and tagRFP-MAP65-2, respectively, was observed in hypocotyl epidermal cells of Arabidopsis by means of ACLSM and/or SIM. **(A)** Overview image from ACLSM (objective 63×/1.40 NA) showing mRFP-TUB6; the boxed area is shown in **(B)**. The white line in **(B)** presents a perpendicular profile, for which normalized intensity measurement is shown in **(C)**. **(D)** Presents quantitative analysis of the resolution of individual TUB6-labeled microtubules by ACLSM. The graph represents averaged, coaligned, and normalized intensity profiles (*N* = 35; FWHM – full-width at half maximum). **(E)** Overview image from ACLSM (objective 63×/1.40 NA) showing tagRFP-MAP65-2; the boxed area is displayed in **(F)**. The white line in **(F)** presents a perpendicular profile, for which normalized intensity measurement is shown in **(G)**. **(H)** Presents quantitative analysis of the resolution of MAP65-2-labeled microtubule bundles by ACLSM. The graph represents averaged, coaligned, and normalized intensity (*N* = 47). **(I)** Overview image from SIM (objective 63×/1.40 NA) showing tagRFP-MAP65-2; the boxed area is displayed in **(J)**. The white line in **(J)** presents a perpendicular profile, for which normalized intensity measurement is shown in **(K)**. **(L)** Presents quantitative analysis of the resolution of MAP65-2-labeled microtubule bundles by SIM. The graph represents averaged, coaligned, and normalized intensity profiles (*N* = 43). Scale bars = 2 μm.

FIGURE S2 Comparison of resolving details of cortical microtubules or MAP65-2 decoration of cortical microtubules by employing TUA6-GFP, mRFP-TUB6, eGFP-MAP65-2 or tagRFP-MAP65-2 and by the means of ACLSM or SIM platform. **(A–D)** Boxplots present the full-width at half maximum (FWHM) values. **(A)** Comparison of TUA6-GFP and mRFP-TUB6 imaged by ACLSM. **(B,C)** Resolution reached by either ACLSM **(B)** or SIM **(C)** for eGFP-MAP65-2 and tagRFP-MAP65-2. **(D)** Comparison of resolution reached by ACLSM or SIM for tagRFP-MAP65-2. **(A–E)** For statistical analysis, Mann–Whitney *U* test was used (^∗∗∗^ significant at *p* 0.001, ns, not statistically significant) and the results are in **(E)**, where M is median and U is result of Mann–Whitney test. Description of box plot: average is represented by ×, median by the middle line, 1st quartile by the bottom line, 3rd quartile by the top line; the whiskers lie within the 1.5× interquartile range (defined from the 1st to the 3rd quartile), outliers are marked by points.

TABLE S1 List of primers used for cloning.

VIDEO S1 Full frame ACLSM video corresponding to Figure 7.

VIDEO S2 Full frame 2D SIM video corresponding to Figure 8.

VIDEO S3 Full frame TIRF-SIM video corresponding to Figure 9.
