# Supplementary figures and images for: Complementary Superresolution Visualization of Composite Plant Microtubule Organization and Dynamics
Source: Front Plant Sci. 2020 Jun 5;11:693. doi: 10.3389/fpls.2020.00693 (PMC7290007; doi:10.3389/fpls.2020.00693)

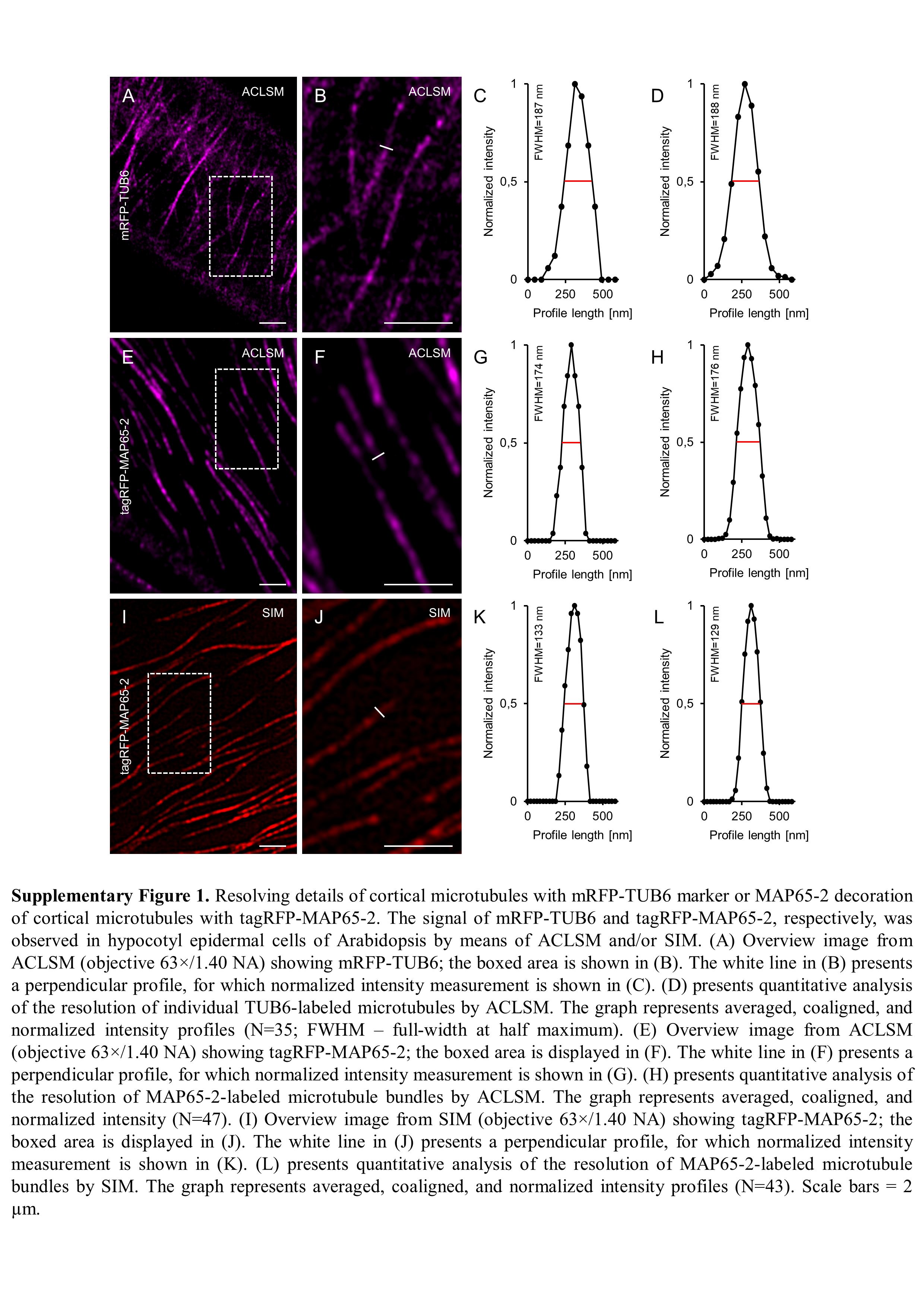

Supplement: FIGURE S1 — Resolving details of cortical microtubules with mRFP-TUB6 marker or MAP65-2 decoration of cortical microtubules with tagRFP-MAP65-2. The signal of mRFP-TUB6 and tagRFP-MAP65-2, respectively, was observed in hypocotyl epidermal cells of Arabidopsis by means of ACLSM and/or SIM. (A) Overview image from ACLSM (objective 63×/1.40 NA) showing mRFP-TUB6; the boxed area is shown in (B). The white line in (B) presents a perpendicular profile, for which normalized intensity measurement is shown in (C). (D) Presents quantitative analysis of the resolution of individual TUB6-labeled microtubules by ACLSM. The graph represents averaged, coaligned, and normalized intensity profiles (N = 35; FWHM – full-width at half maximum). (E) Overview image from ACLSM (objective 63×/1.40 NA) showing tagRFP-MAP65-2; the boxed area is displayed in (F). The white line in (F) presents a perpendicular profile, for which normalized intensity measurement is shown in (G). (H) Presents quantitative analysis of the resolution of MAP65-2-labeled microtubule bundles by ACLSM. The graph represents averaged, coaligned, and normalized intensity (N = 47). (I) Overview image from SIM (objective 63×/1.40 NA) showing tagRFP-MAP65-2; the boxed area is displayed in (J). The white line in (J) presents a perpendicular profile, for which normalized intensity measurement is shown in (K). (L) Presents quantitative analysis of the resolution of MAP65-2-labeled microtubule bundles by SIM. The graph represents averaged, coaligned, and normalized intensity profiles (N = 43). Scale bars = 2 μm. [file Image_1.TIF]

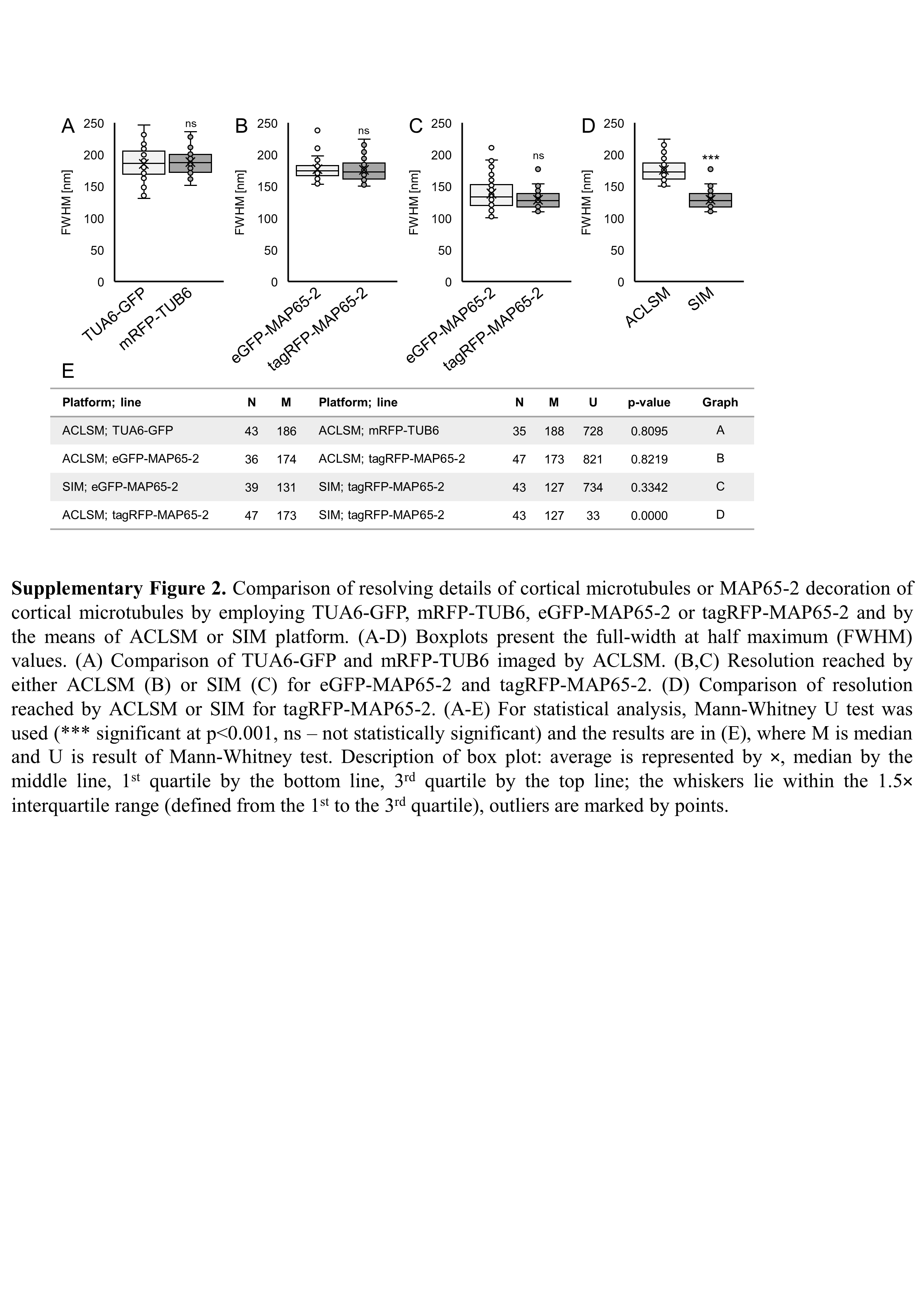

Supplement: FIGURE S2 — Comparison of resolving details of cortical microtubules or MAP65-2 decoration of cortical microtubules by employing TUA6-GFP, mRFP-TUB6, eGFP-MAP65-2 or tagRFP-MAP65-2 and by the means of ACLSM or SIM platform. (A–D) Boxplots present the full-width at half maximum (FWHM) values. (A) Comparison of TUA6-GFP and mRFP-TUB6 imaged by ACLSM. (B,C) Resolution reached by either ACLSM (B) or SIM (C) for eGFP-MAP65-2 and tagRFP-MAP65-2. (D) Comparison of resolution reached by ACLSM or SIM for tagRFP-MAP65-2. (A–E) For statistical analysis, Mann–Whitney U test was used (∗∗∗ significant at p 0.001, ns, not statistically significant) and the results are in (E), where M is median and U is result of Mann–Whitney test. Description of box plot: average is represented by ×, median by the middle line, 1st quartile by the bottom line, 3rd quartile by the top line; the whiskers lie within the 1.5× interquartile range (defined from the 1st to the 3rd quartile), outliers are marked by points. [file Image_2.TIF]
