# Supplementary material for: Complementary Superresolution Visualization of Composite Plant Microtubule Organization and Dynamics
Source: Front Plant Sci. 2020 Jun 5;11:693. doi: 10.3389/fpls.2020.00693 (PMC7290007; doi:10.3389/fpls.2020.00693)
Supplement: TABLE S1 — List of primers used for cloning. [file Table_1.DOCX]

**Supplementary Table 1.** List of primers used for cloning

| **Primer** | **Sequence** |
| --- | --- |
| pGWB502link-F | 5’-ggttaatta atcctagga atctgaagc actgccttg tggtacctg agct-3’ |
| pGWB502link-R | 5’-caggtacca caaggcagt gcttcagat tcctaggat taattaacc tgca-3’ |
| pMAP65-2-F | 5’-cacactagt taattaagg atgaagtaa aggccgaga-3’ |
| pMAP65-2-R | 5’-ggtctaggt accttcttg tgaagtcaa aaggtttcc-3’ |
| MAP65-2cDNA-F | 5’-cacactagg gtaccatgg cagtgacag aagcaga-3’ |
| MAP65-2cDNA-R | 5’-ctctctacg tacgggatc cattgtcac ggtgaagcc atc-3’ |
| eGFP-F | 5’-tcctgtcag gtaccaaga agaaaaatg gtgagcaag ggcgaggag ctgttca-3’ |
| eGFP-R | 5’-cacagtttg gtaccctta gcagctgcc tcttttgcg gcagcctct ttagcagca gcttccttg tacagctcg tccatgccg agagtga-3’ |
| tagRFP-F | 5’-tcctgtcag gtaccaaga agaaaaatg gtgtctaag ggcgaagag ctgattaag g-3’ |
| tagRFP-R | 5’-cacagtttg gtaccctta gcagctgcc tcttttgcg gcagcctct ttagcagca gcttcatta agtttgtgc cccagtttg ctaggga-3’ |
